# Genome-wide comparative analysis of clinical and environmental strains of the opportunistic pathogen *Paracoccus yeei* (*Alphaproteobacteria*)

**DOI:** 10.3389/fmicb.2024.1483110

**Published:** 2024-11-06

**Authors:** Magdalena Szuplewska, Dorota Sentkowska, Robert Lasek, Przemysław Decewicz, Mateusz Hałucha, Łukasz Funk, Cora Chmielowska, Dariusz Bartosik

**Affiliations:** ^1^Department of Bacterial Genetics, Institute of Microbiology, Faculty of Biology, University of Warsaw, Warsaw, Poland; ^2^Department of Environmental Microbiology and Biotechnology, Institute of Microbiology, Faculty of Biology, University of Warsaw, Warsaw, Poland

**Keywords:** *Paracoccus yeei*, opportunistic pathogen, multipartite genome, chromid, evolution of pathogenic bacteria, transposable element, non-autonomous transposable element, urease

## Abstract

**Introduction:**

*Paracoccus yeei* is the first species in the genus *Paracoccus* to be implicated in opportunistic infections in humans. As a result, *P. yeei* strains provide a valuable model for exploring how bacteria shift from a saprophytic to a pathogenic lifestyle, as well as for investigating the role of horizontally transferred DNA in this transition. In order to gain deeper insights into the unique characteristics of this bacterium and the molecular mechanisms underlying its opportunistic behavior, a comparative physiological and genomic analysis of *P. yeei* strains was performed.

**Results:**

Complete genomic sequences of 7 *P. yeei* isolates (both clinical and environmental) were obtained and analyzed. All genomes have a multipartite structure comprising numerous extrachromosomal replicons (59 different ECRs in total), including large chromids of the DnaA-like and RepB families. Within the mobile part of the *P. yeei* genomes (ECRs and transposable elements, TEs), a novel non-autonomous MITE-type element was identified. Detailed genus-wide comparative genomic analysis permitted the identification of *P. yeei*-specific genes, including several putative virulence determinants. One of these, the URE gene cluster, determines the ureolytic activity of *P. yeei* strains—a unique feature among *Paracoccus* spp. This activity is induced by the inclusion of urea in the growth medium and is dependent on the presence of an intact *nikR* regulatory gene, which presumably regulates expression of nickel (urease cofactor) transporter genes.

**Discussion:**

This in-depth comparative analysis provides a detailed insight into the structure, composition and properties of *P. yeei* genomes. Several predicted virulence determinants (including URE gene clusters) were identified within ECRs, indicating an important role for the flexible genome in determining the opportunistic properties of this bacterium.

## 1 Introduction

Bacteria of the genus *Paracoccus* (family *Paracoccaceae*, order *Rhodobacterales*, class *Alphaproteobacteria*) are known for their great metabolic diversity and flexibility. Many of them can switch between growth modes, using different carbon and energy sources, and employing various final electron acceptors (Czarnecki and Bartosik, 2019). There are currently 105 known species of the genus *Paracoccus* (NCBI Taxonomy, 17 July 2024), including *Paracoccus denitrificans*—the type strain of the genus (isolated in 1910) —commonly used as a model denitrifying organism (Beijerinck and Minkman, 1910; Bordel et al., 2024). A large number of these species were identified only recently and many have yet to be the subject of detailed studies.

*Paracoccus* spp. inhabit diverse marine and terrestrial environments, such as soil, sediments, sludge, brines or groundwater (e.g., Urakami et al., 1990; Siller et al., 1996; Tsubokura et al., 1999; Berry et al., 2003; Lee et al., 2004; Liu et al., 2006, 2008). In addition, some strains have been isolated from the rhizosphere or from the surface of other organisms, e.g., ticks, marine bryozoans and corals (Pukall et al., 2003; Ghosh et al., 2006; Carlos et al., 2017). The ubiquity of these bacteria is further demonstrated by their presence in house dust (Thompson et al., 2021).

Among these environmental bacteria, *Paracoccus yeei* (formerly classified as a eugonic oxidizer in group 2; strain EO-2) is of particular interest because it is the first species of the genus *Paracoccus* to be implicated in opportunistic infections in humans (Daneshvar et al., 2003). This bacterium (naturally occurring in soil) is not associated with a specific disease (Fosso et al., 2021). *P. yeei* isolates have been recovered from several clinical conditions, e.g., from the dialysate of a patient with peritonitis, myocarditis in a transplanted heart, corneal transplantation, bacteremia, keratitis, otitis and dermatological lesions (Lasek et al., 2018). The number of cases of infection by *P. yeei* appears to be increasing (Funke et al., 2004; Kanis et al., 2010; Schweiger et al., 2011; Courjaret et al., 2014; Arias and Clark, 2019; Aliste-Fernández et al., 2020; Fosso et al., 2021; Shifera et al., 2021; Bhikoo et al., 2022). However, this reported incidence is likely to be an underestimate since current common diagnostic tests do not detect this bacterium (Sack et al., 2017). Moreover, *P. yeei* cells can appear under-decolorized after Gram staining, so may be inadvertently reported or dismissed as gram-positive cocci (Dyer and Harris, 2020).

Analysis of a larger number of clinical cases suggests that immunocompromised patients are at increased risk of infection with this bacterium (Dyer and Harris, 2020). However, the natural reservoir of this opportunistic pathogen as well as the molecular bases of its pathogenicity remain unclear.

Numerous *P. yeei* strains have been isolated from everyday objects in hospital environments, which may play an important role in its transmission. This bacterium was found on the surface of medical equipment, such as operating tables, endoscopes or laryngoscopes (in direct contact with the mucosa, saliva and blood of patients) (Choi et al., 2017; Sartoretti et al., 2017; Pasquale et al., 2021), as well as on the surface of mobile phones of hospital employees and patients (Murgier et al., 2016; Cantais et al., 2020). Moreover, in non-hospital settings *P. yeei* strains were detected on the surface of benches, doors and walls of schools and kindergartens (Kruszewska et al., 2021), and on hand-drying devices in public toilets (Huesca-Espitia et al., 2018). Interestingly, this bacterium was also identified as a dominant member of microbial consortia contributing to the biodegradation of pre-Columbian canvasses in a museum collection (Pietrzak et al., 2017).

*P. yeei* represents a convenient model organism to study the switch from a saprophytic to a pathogenic lifestyle and to determine the role of horizontally acquired DNA in this process. Comparative genomic analyses of *P. yeei* and other members of the genus *Paracoccus* should yield a great deal of information on the mechanisms involved in this transition. Our previous analysis of the genomes of 3 *P. yeei* isolates (CCUG 32053, FDAARGOS_252 and TT13) gave the first insight into the genome composition, mobilome and metabolic potential of these bacteria (Lasek et al., 2018). However, the small number of strains analyzed was a major limitation of this study.

The main objective of the present study was to define the specific properties and genetic information of *P. yeei* species that are potentially involved in the process of pathogenesis, as well as to characterize mobile genetic elements—natural gene carriers that may be responsible for the horizontal transmission of virulence determinants. For 7 *P. yeei* strains available in our laboratory, we performed various physiological tests and detailed analyses of their mobile genetic elements. To identify *P. yeei*-specific genes, we conducted a comparative analysis of the 11 genomes of *P. yeei* strains available in the NCBI database (both clinical and environmental isolates) and four genomes of other members of the genus *Paracoccus*.

## 2 Materials and methods

### 2.1 Strains and culture conditions

*P. yeei* strains CCUG 13493, CCUG 17731, CCUG 32052, CCUG 32054, CCUG 46822, and CCUG 54214 were purchased from the Culture Collection of the University of Gothenburg (CCUG) (Sweden). Strain LM20 was isolated from black shale collected within the Lubin underground copper mine in Poland (Dziewit et al., 2015). *P. yeei* CCUG 13493R, CCUG 17731R, CCUG 32052R, CCUG 32054R, CCUG 46822R, CCUG 54214R, and LM20R, rifampicin-resistant derivatives of the respective wild-type strains, and *Escherichia coli* TG1 (Sambrook and Russell, 2001), were used as plasmid recipients. *E. coli* DH5α (Hanahan, 1983) was the host strain of helper plasmid pRK2013, used in triparental matings (Ditta et al., 1980) (Supplementary Table S1).

All strains were cultured in lysogeny broth (LB) medium (Sambrook and Russell, 2001) at 37°C for *E. coli* or 30°C for the other strains. Liquid cultures were grown under shaking conditions. When required, the medium was supplemented with 10% sucrose and the following concentrations of selective antibiotics: kanamycin, 50 μg/ml for all strains except *Achromobacter* sp. LM16R (300 μg/ml) (Dziewit et al., 2015); rifampicin, 50 μg/ml; tetracycline, 2 μg/ml for *P. yeei* and 20 μg/ml for *E. coli* strains.

### 2.2 Physiological analyses

The response to various growth conditions (temperature, pH and salinity) of *P. yeei* strains was determined as described previously by Dziewit et al. (2013). Motility was tested according to the method previously outlined by Dziewit et al. (2015). The hemolytic ability of different strains was tested by growth on blood agar plates that were incubated at 30 and 37°C under aerobic conditions—the results were read after 24 and 48 h of incubation. Siderophore production was assessed using the chrome azurol S (CAS) agar plate method, as described previously (Dziewit et al., 2015). The plates were incubated in the dark at 30°C for 72 h, after which halo formation around the colonies was assessed. Each isolate was phenotypically characterized using (i) the API 50 CH test system (to differentiate strains based on their ability to metabolize different carbohydrates) and (ii) the API ZYM test system (to detect and identify enzymatic activities); according to the recommendations of the supplier (bioMerieux, Marcy l'Etoile, France). Ureolytic activity was detected by cultivation on Christensen's urea medium, containing urea and a pH indicator (phenol red) (Christensen, 1946) or using rapid urease tests. *Proteus vulgaris* and/or *Klebsiella pneumoniae* strains (University of Warsaw collection) were used as positive controls, and *P. aminophilus* JCM 7686 and/or *P. aminovorans* JCM 7685 as negative controls (Urakami et al., 1990). The minimal inhibitory concentrations (MIC) of antimicrobial agents were determined for *P. yeei* strains by using Mueller Hinton II agar diffusion with E-tests as recommended by the supplier (bioMérieux). *E. coli* ATCC 25922 was used as a control. To confirm reproducibility, antimicrobial susceptibility testing was repeated at least twice. Since no interpretative guidelines for *P. yeei* susceptibility have been published, we used the criteria proposed by Arias and Clark (2019). MIC values for selected heavy metal ions were established on titration plates using a procedure described previously (Dziewit et al., 2013). The following heavy metal salts were used to prepare appropriate stock solutions in water: NaAsO_2_; 3CdSO_4_ × 8H_2_O; CoSO_4_ × 7H_2_O; K_2_Cr_2_O_7_; CuSO_4_; HgCl_2_; NiCl_2_ × 6H_2_O; NaO_3_V; ZnSO_4_ × 7H_2_O. Each microplate was monitored for growth using an automated microplate reader at 24-h intervals for 3 days. Isolates that demonstrated growth at the following minimum metal ion concentrations were classified as resistant using previously described criteria (Dziewit et al., 2015): (i) 20 mM V^5+^, (ii) 1 mM As^3+^, Cd^2+^, Co^2+^, Cu^2+^, Ni^2+^, Zn^2+^ or Cr^6+^, and (iii) 0.1 mM Hg^2+^.

### 2.3 DNA isolation, standard molecular biology procedures and PCR conditions

Plasmid DNA was isolated using the alkaline lysis procedure (Birnboim and Doly, 1979) or purified by CsCl-ethidium bromide gradient centrifugation (Sambrook and Russell, 2001). The visualization of mega-sized replicons was achieved by in-gel lysis and DNA electrophoresis (Eckhardt, 1978; Hynes and McGregor, 1990). Plasmid DNA was also isolated using a Plasmid Mini kit (A&A Biotechnology), GeneMATRIX Miniprep DNA Purification Kit (EUR_x_) and GeneJET Plasmid Miniprep Kit (Thermo Fisher Scientific). Routine DNA manipulation was performed using standard methods (Sambrook and Russell, 2001). DNA amplification by PCR was performed in a Mastercycler (Eppendorf) using synthetic oligonucleotides (Supplementary Table S1), High Fidelity Taq DNA polymerase (Qiagen) or Phusion Hi-Fidelity DNA polymerase (Thermo Fisher Scientific), dNTPs and appropriate template DNAs, as described previously (Bartosik et al., 2003).

### 2.4 Introduction of plasmid DNA into bacterial cells

Chemical transformation of *E. coli* cells was performed by a standard method (Kushner, 1978). Plasmid DNA was introduced into *Paracoccus* spp. strains by triparental mating using helper *E. coli* strain DH5α carrying plasmid pRK2013 (containing the transfer system of plasmid RP4) (Ditta et al., 1980), as described previously (Lasek et al., 2018).

### 2.5 Plasmid host range testing

REP systems of extrachromosomal replicons of the analyzed strains were cloned in vector pABW1 (Bartosik et al., 1997) which cannot replicate in *Paracoccus* strains or other tested recipients (Supplementary Table S1). These shuttle plasmid constructs were introduced into the following strains: (i) class *Alphaproteobacteria*—*Agrobacterium tumefaciens* LBA 288R (Hooykaas et al., 1980), *Paracoccus aminophilus* JCM 7686R (without plasmids pAMI2, pAMI3) (Dziewit et al., 2014), *Paracoccus aminovorans* JCM 7686R (Czarnecki et al., 2017), *Paracoccus pantotrophus* KL100 (Jordan et al., 1997), (ii) class *Betaproteobacteria*—*Achromobacter* sp. LM16R (Dziewit et al., 2015) and (iii) class *Gammaproteobacteria*—*Cronobacter sakazakii* ATCC 29544 (Iversen et al., 2008) and *Pseudomonas* sp. LM6R (Dziewit et al., 2015).

### 2.6 Identification of functional transposable elements

Trap plasmids pMAT1 and pMEC1 (Bartosik et al., 2003; Szuplewska and Bartosik, 2009; Gay et al., 1985; Schneider et al., 2000) were used for the identification of functional TEs of *P. yeei* (Supplementary Table S1). These plasmids were transferred from *E. coli* TG1 to *P. yeei* strains by triparental mating. The “capture” of TEs was verified by PCR using primers specific to the selection cassettes, with DNA isolated from Tc^r^ and Suc^r^ mutants as the template (Supplementary Table S1). A total of 1,304 clones were tested this way. The amplified DNA fragments were subjected to electrophoretic analysis, which permitted the detection of DNA elements (TEs), ranging in size from ~0.8 to 1.5 kb, embedded in the cassettes. DNA sequencing of the termini of individual TEs was used to identify the complete elements within the genome sequences of their parental strains. All trapped TEs were sequenced and compared with the ISfinder database (Siguier et al., 2006).

### 2.7 Mutational analysis of URE modules

To confirm that the identified URE modules are responsible for ureolytic activity, mutational analysis was performed using the gene replacement method. Mutations (insertion of a Km^r^ cassette) were introduced into URE type 1 modules, either in the 5′ region of the Ureα subunit gene or within the *nikR* gene. Both mutations were created in the strain CCUG 32053 (which contains only URE type 1) and the strain CCUG 13493 (which also contains a URE type 2 module). The following procedure was used to obtain *ure*α::Km^r^ and *nikR*::Km^r^ mutants: (i) DNA fragments containing *ure*α*-ure*β*-ure*γ or part of the region encoding NikR and nickel transporters were amplified by PCR using CCUG 32053 DNA as the template, (ii) these DNA fragments were cloned into the SacI and SphI sites of the mobilizable suicide vector pDS132 (Philippe et al., 2004), (iii) the Km^r^ cassette from plasmid pDIY-Km (Dziewit et al., 2011) was inserted into the AleI site within these fragments to obtain constructs pDS132-*ure*α and pDS132-*nikR* (Supplementary Table S1); (iv) these plasmids were then introduced into strains CCUG 32053R and CCUG 13493R by conjugation. Mutant clones *ure*α::Km^r^ and *nikR*::Km^r^ were selected on medium supplemented with kanamycin (for recombinant selection) and sucrose (for counter-selection). The introduced mutations were confirmed by DNA sequencing. Ureolytic activity of the obtained mutants was tested following growth on minimal medium supplemented with 1% urea, using rapid urease tests. Complementation of the mutants was performed using plasmids pBBR-*ure*α or pBBR-*nikR*, which were prepared by cloning PCR fragments containing *ure*α*-ure*β*-ure*γ or part of the region encoding NikR and nickel transporters into vector pBBR-MCS5 (Gm^r^) (Kovach et al., 1995) (Supplementary Table S1).

### 2.8 Genome sequencing

The *P. yeei* genomes were sequenced using a combination of Oxford Nanopore and Illumina technologies, as described previously (Lasek et al., 2018).

### 2.9 Bioinformatic analyses

Sequence annotation and bioinformatic analyses (identification of tRNA genes and rRNA operons, relaxases—MOB, toxin-antitoxin modules, transposable elements—TE) were performed as described previously (Lasek et al., 2018). Potential ICE and IME elements were predicted using ICEfinder (Liu et al., 2019).

Categories for clusters of orthologous groups (COGs) were assigned to each protein by performing a local RPS-BLAST search against the COG database (last updated on January 22, 2015). A threshold e-value of 1e-5 was used, and only the top BLAST hits were taken into account (Tatusov et al., 2003).

The core genome of *Paracoccus* spp. and *P. yeei* species was defined based on the complete genomic sequences of *P. aminophilus* JCM 7686 (Dziewit et al., 2014), *P. aminovorans* JCM 7685 (Czarnecki et al., 2017), *P. contaminans* RKI16-01929T (Aurass et al., 2017) and *P. denitrificans* PD1222 (NC_008686-8), and eleven *P. yeei* strains: CCUG 32053 (Lasek et al., 2018), FDAARGOS_252 (NZ_CP020440–47), FDAARGOS_643 (NZ_CP044078-82), TT13 (Lim et al., 2018) and CCUG 13493, CCUG 17731, CCUG 32052, CCUG 32054, CCUG 46822, CCUG 54214, LM20 (Dziewit et al., 2015). Proteins encoded within the genomes were used in all-against-all BLASTp searches utilized by a stand-alone version of OrthoVenn2 (Xu et al., 2019), using an e-value of 1e-15 as the threshold and inflation level of 1.5. Based on this analysis, all proteins were clustered into groups reflecting similarity and designated as core proteins when encoded by all eleven genomes or as singletons when the respective gene was identified in only a single genome. A similar approach with a different manner of grouping was applied to identify strain-specific gene clusters.

Protein datasets from the Virulence Factor Database VFDB (Chen et al., 2016), the Comprehensive Antibiotic Resistance Database CARD (Alcock et al., 2020) and Virulence Factors Database VICTORS (Sayers et al., 2019), all downloaded on April 1 2021, were used for BLASTp searches (e-value threshold of 1e-30 and 80% of query coverage per HSP) with the proteomes of *P. yeei* and four other *Paracoccus* spp. strains listed above to identify putative *P. yeei* species virulence determinants.

EasyFig (Sullivan et al., 2011) was used to perform comparative genomic analyses (including genomes and plasmidomes comparisons) and visualize the results. RNA secondary structures were predicted by *in silico* folding using Mfold software (Zuker, 2003).

Phylogenetic analysis of the genus *Paracoccus* was based on alignment of concatenated nucleotide sequences of selected core genes: *atpD, dnaA, dnaK, gyrB, recA, rpoB*, and *thrC* (homologous genes of *Roseobacter denitrificans* OCh 114 were used as an outgroup), as described previously (Lasek et al., 2018). Nucleotide alignments were obtained with Mafft (Katoh et al., 2019). Then, concatenated genes were analyzed with ModelTest-NG (Darriba et al., 2020), checking all models to select the best-fit nucleotide substitution model. The selected substitution model was applied in RaxML-NG (Kozlov et al., 2019) with 2,000 regular bootstrap replicates performed on the best Maximum Likelihood (ML) tree selected from 100 independently generated ML starting trees.

### 2.10 Nucleotide sequence accession numbers

The nucleotide sequences of the *P. yeei* chromosomes and extrachromosomal replicons were deposited in GenBank (NCBI), with the following accession numbers: (i) CCUG 13493—CP038080 (chromosome), CP038073 (plasmid pYEE13493P1), CP038074 (pYEE13493P2), CP038075 (pYEE13493P3), CP038076 (pYEE13493P4), CP038077 (pYEE13493P5), CP038078 (pYEE13493P7), CP038079 (pYEE13493P8), CP038081 (pYEE13493P6); (ii) CCUG 17731—CP038042 (chromosome), CP038035 (pYEE17731P1), CP038036 (pYEE17731P2), CP038037 (pYEE17731P3), CP038038 (pYEE17731P4), CP038039 (pYEE17731P5), CP038040 (pYEE17731P6), CP038041 (pYEE17731P7); (iii) CCUG 32052—CP038090 (chromosome), CP038082 (pYEE32052P1), CP038083 (pYEE32052P2), CP038084 (pYEE32052P3), CP038085 (pYEE32052P4), CP038086 (pYEE32052P5), CP038087 (pYEE32052P6), CP038088 (pYEE32052P7), CP038089 (pYEE32052P8); (iv) CCUG 32054—CP038095 (chromosome), CP038091 (pYEE32054P6), CP038092 (pYEE32054P7), CP038093 (pYEE32054P8), CP038094 (pYEE32054P9), CP038096 (pYEE32054P1), CP038097 (pYEE32054P2), CP038098 (pYEE32054P3), CP038099 (pYEE32054P4), CP038100 (pYEE32054P5); (v) CCUG 46822—CP038056 (chromosome), CP038043 (pYEE46822P1), CP038044 (pYEE46822P2), CP038045 (pYEE46822P3), CP038046 (pYEE46822P4), CP038047 (pYEE46822P5), CP038048 (pYEE46822P6), CP038049 (pYEE46822P7), CP038050 (pYEE46822P8), CP038051 (pYEE46822P9), CP038052 (pYEE46822P10), CP038053 (pYEE46822P11), CP038054 (pYEE46822P12), CP038055 (pYEE46822P13); (vi) CCUG 54214—CP038061 (chromosome), CP038057 (pYEE51214P1), CP038058 (pYEE51214P2), CP038059 (pYEE51214P3), CP038060 (pYEE51214P4); (vii) LM20—CP038072 (chromosome), CP038062 (pLM20P1), CP038063 (pLM20P2), CP038064 (pLM20P3), CP038065 (pLM20P4), CP038066 (pLM20P5), CP038067 (pLM20P6), CP038068 (pLM20P7), CP038069 (pLM20P8), CP038070 (pLM20P9), CP038071 (pLM20P10). The nucleotide sequences of newly identified ISs (IS*Pye*73—IS*Pye*80) and MITE*Pye1* were deposited in the ISfinder database (Siguier et al., 2006).

## 3 Results

### 3.1 *P. yeei* strains selected for characterization

Seven (five clinical and two environmental) isolates of *P. yeei* were selected for detailed characterization (Table 1). Clinical strains were sourced from two continents—they were isolated in different geographical locations, at different times and from different clinical cases (Table 1). The other strains were isolated either from an anthropogenic environment (CCUG 54214; a metal surface), or from the natural environment (LM20; organic-rich black shale from the Lubin copper mine in Poland; Dziewit et al., 2015). The clinical isolate CCUG 32053, characterized in a previous study (Lasek et al., 2018), was used as a reference strain.

**Table 1:** *Paracoccus yeei* strains analyzed in this study.

***P. yeei* strain**	**Source of isolation**			**References**
	**Specimen**	**Locality**	**Year**	
**Clinical isolates (human)**				
CCUG^a^ 13493	Foot wound	USA (Virginia)	1980	–
CCUG 17731	Water, hemodialysis	France (Grenoble)	1985	–
CCUG 32052	Cerebrospinal fluid	USA (Puerto Rico)	1983	–
CCUG 32053^b^	Eye	USA (Missouri)	1981	Lasek et al., 2018
CCUG 32054	Facial sinus	USA (Washington)	1985	–
CCUG 46822	Abdominal dialysate	USA (Pennsylvania)	1988	Daneshvar et al., 2003
**Environmental isolates**				
CCUG 54214	Metal product, industry	Sweden	2007	–
LM20	Black shale (copper mine)	Poland (Lubin)	2015	Dziewit et al., 2015

^a^*P. yeei* strains with the designation CCUG were purchased from the Culture Collection of the University of Gothenburg (CCUG) (Sweden).

^b^Reference strain.

#### 3.1.1 Physiological and phenotypic characterization

*P. yeei* is a non-motile, oxidase- and catalase-positive gram-negative bacterium. The analyzed strains were facultative aerobes capable of nitrate reduction and denitrification (except for CCUG 32052). The strains were mesophilic, capable of growth at temperatures ranging from 21 to 37°C (except LM20, which grew from 15 to 37°C) (Table 2). All strains grew in LB medium at pH values close to 7 (ranging from 5 to 8 or 9), typical for neutrophilic bacteria. Salinity tolerance testing showed that the isolates could tolerate NaCl concentrations of 4–6%. These bacteria were non-hemolytic, did not produce bacteriocins, and exhibited weak adhesion to polystyrene. However, all produced siderophores, as determined by the chrome azurol S (CAS) agar plate assay. They demonstrated the ability to degrade 27 carbohydrates (out of 49 tested), with identical profiles for all strains except LM20, which is unable to ferment mannitol, sorbitol or arabitol (Table 2). The enzymatic capabilities of these strains were assessed by testing for 19 hydrolytic enzymes from various groups. All strains produced alkaline phosphatase (except CCUG 13493), esterase, lipase esterase, leucine arylamidase, acid phosphatase, naphthol-AS-BI phosphohydrolase, and α-glucosidase, with only CCUG 17731 producing β-galactosidase (Table 2). The strains also displayed ureolytic activity, which is a unique feature among *Paracoccus* spp.

**Table 2:** Phenotypic and physiological characteristics of *P. yeei* strains.

**Characteristic(s)**	***P. yeei*** **clinical isolates**					**Environmental isolates**	
	**CCUG 13493**	**CCUG 17731**	**CCUG 32052**	**CCUG 32054**	**CCUG 46822**	**CCUG 54214**	**LM20** ^a^
	**General features**						
Temperature range for growth (°C, optimum)	21–37 (30)	21–37 (30)	21–37 (30)	21–37 (30)	21–37 (30)	21–37 (30)	15–37 (30)
NaCl tolerance (%)	6	4	4	6	4	6	4
pH range for growth	5–9	5–8	5–9	5–8	5–8	5–9	4–9
Motility	□	□	□	□	□	□	□
Hemolysis	□	□	□	□	□	□	□
Siderophore production	■	■	■	■	■	■	■
	**Nitrate metabolism**						
Nitrate reduction	■	■	■	■	■	■	■
Gas from nitrate	■	■	□	■	■	■	■
	**Carbohydrate fermentation (API 50 CH test)**						
D-Arabinose, L-arabinose, D-ribose, D-xylose, L-xylose, D-galactose, D-glucose, D-fructose, D-mannose, L-sorbose, L-rhamnose, dulcitol, inositol, D-cellibiose, D-lactose, D-melibiose, xylitol, gentibiose, D-lyxose, D-tagatose, D-fucose, L-fucose, L-arabitol	■	■	■	■	■	■	■
D-Adonitol	■	■	■	■	■	■	■
D-Mannitol, D-sorbitol, D-arabitiol	■	■	■	■	■	■	□
Glycerol, erythritol, methyl-βD-xylopyranoside, methyl-αD-mannopyranoside, methyl-αD-glucopyranoside, N-acetylglucosamine, amygdaline, arbutine, esculine, salicin, D-maltose, D-sacharose, D-trehalose, inulin, D-melezitose, D-raffinose, amidon, glycogen, D-turanose, potassium gluconate, potassium 2 ketogluconate, potassium 5 ketogluconate	□	□	□	□	□	□	□
	**Enzyme activities (including API ZYM test)**						
Esterase, esterase lipase, leucine arylamidase, acid phosphatase, naphthol-AS-BI-phosphohydrolase,α-glukosidase	■	■	■	■	■	■	■
Alkaline phosphatase	□	■	■	■	■	■	■
β-Galactosidase	□	■	□	□	□	□	□
Lipase, valine arylamidase, cystine arylamidase, trypsin, α-chymotrypsin, α-galactosidase, β-glucuronidase, β-glucosidase, N-acetyl-β-glucosamidase, α-mannosidase, α-fucosidase	□	□	□	□	□	□	□
Catalase	■	■	■	■	■	■	■
Oxidase	■	■	■	■	■	■	■
Urease activity	■	■	■	■	■	■	■

^a^Growth conditions (temp. range, pH range, NaCl tolerance) for strain LM20 were as described previously (Dziewit et al., 2015).

Filled square ■ indicates a positive result, empty square □ indicates a negative result.

MIC values for selected antibiotics (ampicillin, ciprofloxacin, erythromycin, gentamicin, tetracycline, and vancomycin) were determined for each strain (Supplementary Table S2). The data were analogous to those from previous studies of clinical isolates of *P. yeei*, showing sensitivity to beta-lactams, especially aminopenicillins, as well as macrolides and aminoglycosides (Funke et al., 2004; Wallet et al., 2010; Schweiger et al., 2011; Sastre et al., 2016; Arias and Clark, 2019; Aliste-Fernández et al., 2020). Nevertheless, we observed variations in the susceptibility of the isolates, e.g., CCUG 13493 was most sensitive to the tested antibiotics, CCUG 32054 was the most tolerant, and some strains (CCUG 32052, CCUG 32054, CCUG 46822, LM20) were less sensitive to ciprofloxacin (Supplementary Table S2).

MIC values for selected heavy metal ions were similar for all strains, with no major differences between clinical and environmental isolates. CCUG 54214 showed higher tolerance to As^3+^, Ni^2+^, V^5+^, and Zn^2+^, while LM20 showed higher tolerance to As^3+^, Cd^2+^, and Hg^2+^, reflecting the adaptation of these strains to the environments from which they were isolated (Supplementary Table S2).

### 3.2 Genomic features

**Figure 1:**
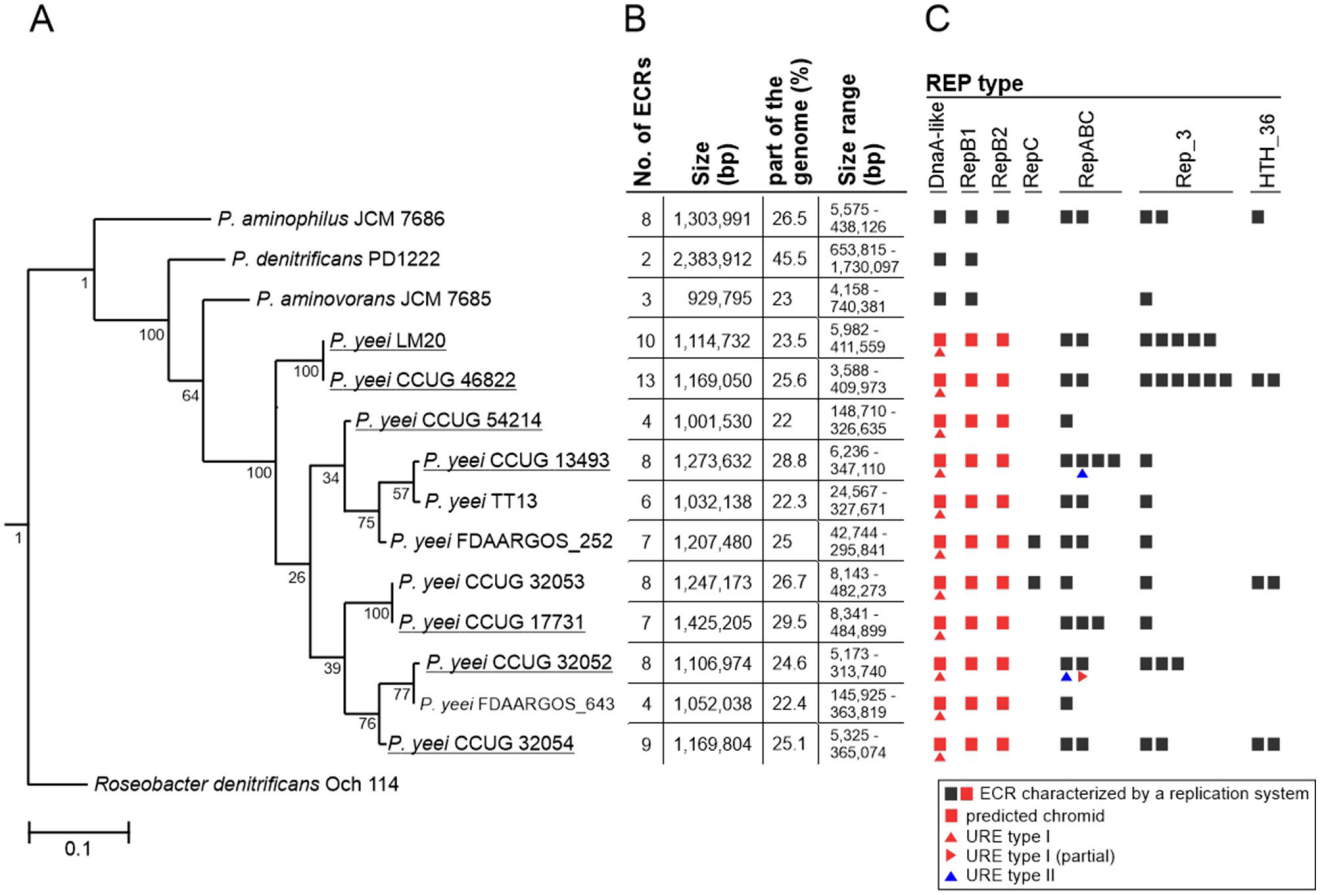
Distribution of ECRs in *P. yeei* genomes. **(A)** Phylogenetic tree of *P. yeei* constructed using core gene nucleotide sequences. Three other members of the genus *Paracoccus*—*P. aminophilus* JCM 7686, *P. aminovorans* JCM 7685, and *P. denitrificans* PD1222—as well as *Roseobacter denitrificans* Och 114 (as an outgroup) were included in the analysis. Bar represents 0.1 nucleotide substitutions per position. **(B)** Summary of data for ECRs of the analyzed *Paracoccus* spp. strains. **(C)** Distribution of different REP module types among ECRs of *Paracoccus* spp.

The genomes of the seven strains of *P. yeei* were fully sequenced and characterized. Their genome sizes range from 4,423,927 to 4,829,807 bp (average 4,610,003 bp). All are multipartite, containing from 4 (CCUG 54214) to 13 (CCUG 46822) extrachromosomal replicons (ECRs), that range in size from 3.5 kb (pYEE46822P1 of CCUG 46822) to 485 kb (pYEE17731P7 of CCUG 17731) (Table 3; Figure 1B; Supplementary Figure S1). In total, the strains contain 59 ECRs (11,665,529 bp), which on average constitute ~25% of the size of each genome (Table 3; Figure 1B). The physicochemical parameters of the genome sequences were determined, including the %GC content (average 67%), the number of protein-coding sequences (CDSs) (average 4,200) and pseudogenes (average 190). The GC content of the ECRs ranges from 53% (pYEE46822P4, CCUG 46822) to 69% (pYEE13493P5, CCUG 13493). The number of CDSs present in ECRs ranges from 849 (CCUG 54214), accounting for 20% of the entire genome, to 1,270 (CCUG 17731) (28%) (Supplementary Table S3). The rRNA operons and most of the tRNA genes are localized within the chromosomes, although some additional tRNA genes (2–3) were identified within ECRs (Table 3).

**Table 3:** General features of *P. yeei* genomes.

**General features**	**Clinical isolates**					**Environmental isolates**	
	**CCUG 13493**	**CCUG 17731**	**CCUG 32052**	**CCUG 32054**	**CCUG 46822**	**CCUG 54214**	**LM20**
Genome size (bp)	4,423,927	4,829,807	4,505,223	4,652,732	4,562,823	4,554,217		4,741,293
ECRs	8	7	8	9	13	4	10
Total ECR size (bp) (% of entire genome)	1,273,632 (28.8)	1,425,205 (29.5)	1,106,974 (24.6)	1,169,804 (25.1)	1,169,050 (25.6)	1,001,530 (22)	1,114,732 (23.5)
**G** + **C content (%)**							
Genome	67.6	67.3	67.4	67.5	67.4	67.5	67.3
ECRs (range)	62.8–69	56.9–68.3	58.5–68.4	60.6–68.5	53.5–68.9	64.5–68.5	57.8–68.9
**CDSs**							
Genome	4,066	4,465	4,168	4,284	4,152	4,162	4,353
ECRs (% of entire genome)	1,117 (27.5)	1,270 (28.4)	964 (23.1)	1,021 (23.8)	998 (24)	849 (20.4)	957 (22)
**Pseudogenes**							
Genome	195	205	179	163	208	188	217
ECRs (%)	96 (49.2)	100 (48.8)	78 (43.6)	74 (45.4)	98 (47.1)	67 (35.6)	95 (43.8)
**RNA genes**							
tRNA genes in genome	50	51	51	51	50	50	50
tRNA genes in ECRs	2	3	2	3	2	2	2
rRNA operons^a^	3	3	3	3	3	3	3
**Integrative elements**							
Predicted prophage regions^b^	2	5	5	4	3	6	7
Predicted ICE/IME elements^c^								2	3	0	2	0	2	1
**Transposase genes** ^d^														
Genome	119	145	150	84	136	125	150
ECRs (% of entire genome)	57 (48)	64 (44)	66 (44)	32 (38)	56 (41)	40 (32)	52 (35)

^a^16S-23S-5S rRNA operons.

^b^Phastest predicted prophage regions (complete) (Supplementary Table S4).

^c^ICEfinder predicted ICE/IME elements (Supplementary Table S4).

^d^Including truncated forms.

### 3.3 Extrachromosomal replicons

**Figure 2:**
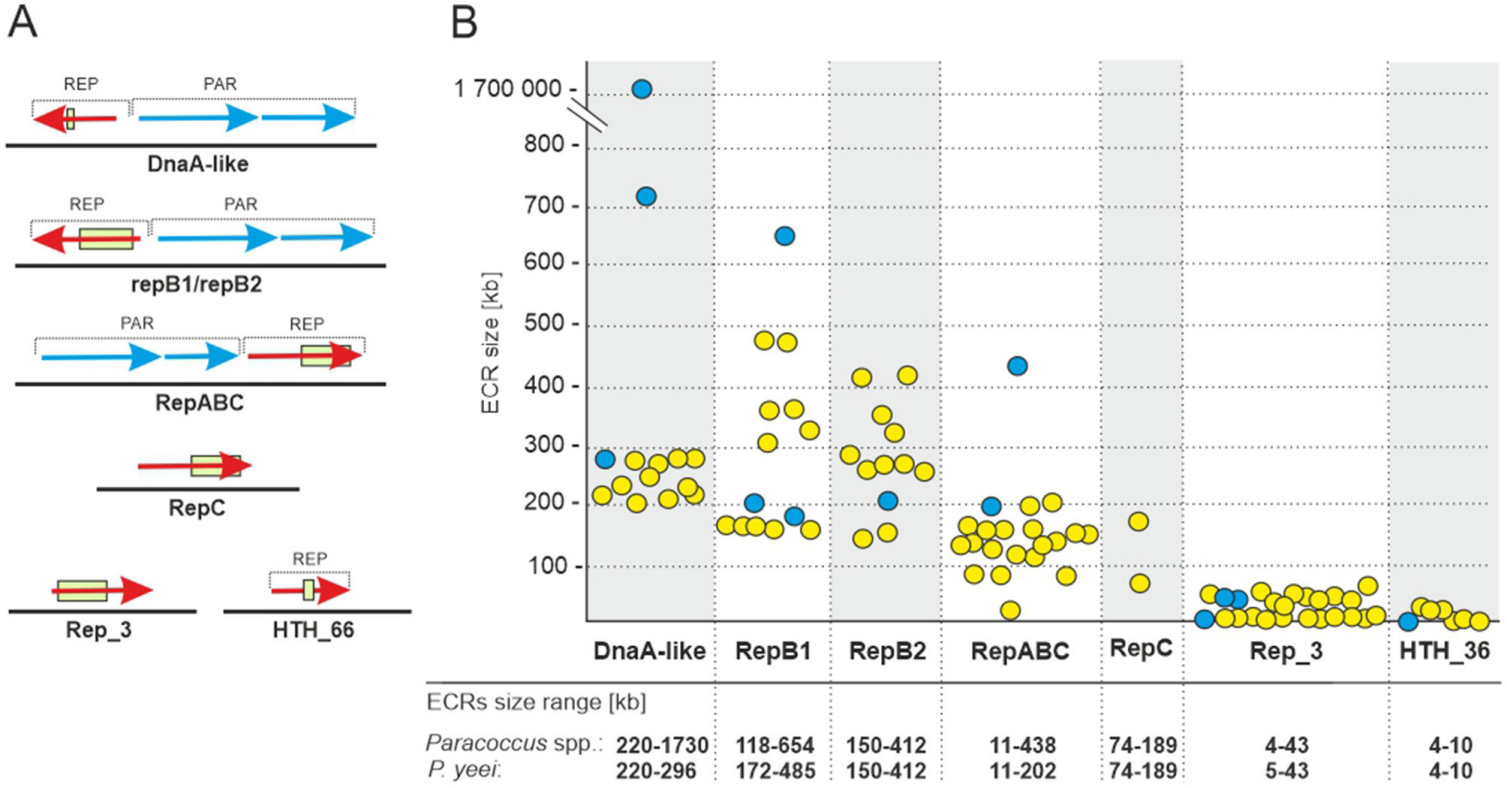
ECRs identified in the genomes of *P. yeei* strains. **(A)** Genetic organization of the replication (REP) and partitioning modules of the ECRs. The box indicates the location of sequence motifs specific to each type of replication initiation protein. **(B)** Size range of *P. yeei* replicons carrying a given REP module type (yellow). The blue color indicates replicons present in the genomes of *P. aminophilus* JCM 7686, *P. aminovorans* JCM 7685, and *P. denitrificans* PD1222.

The analyzed *P. yeei* strains contain numerous ECRs (59 in total)—from 4 (CCUG 54214) to 13 (CCUG 46822) (Figure 1B; Supplementary Figure S1). These are mainly large DNA molecules, constituting a significant proportion of their host strain genomes—from 22% (CCUG 54214) to >28% (CCUG 13493) (Figure 1B). The replication system (REP) and the gene encoding the replication initiation protein (Rep) within each ECR were identified. The REP modules (and entire plasmids) were classified based on the presence of specific amino acid (aa) sequence signatures in the Rep proteins (Figure 2A). Replicons of the DnaA-like, RepB (RepB1 and RepB2), RepABC, RepC, Rep_3 and HTH_36 families were identified, all of which are common among *Paracoccus* spp. and other *Alphaproteobacteria* (Figures 1C, 2).

The ECRs carrying REPs of a given type are generally similar in size, with the exception of RepB1 and RepB2 replicons, in which greater size variation is observed (Figure 2B). The REPs of the DnaA-like, RepB and RepABC types (occurring in each *P. yeei* strain) are characteristic for large replicons, which is consistent with previous observations (e.g., Lasek et al., 2018). Detailed characterization of individual ECRs, including their size, nucleotide sequence GC content, coding capacity and tRNA gene distribution, is presented in Supplementary Table S3.

To test the host range of individual ECRs representing the different replicon families, their REP regions were cloned in mobilizable shuttle plasmids and introduced by triparental mating into recipient strains from different classes of *Proteobacteria* (listed in Section 2). This analysis revealed that all of the analyzed REPs function solely in *Alphaproteobacteria*, so have a relatively narrow host range.

The *P. yeei* ECRs contain numerous *loci* enabling their stable maintenance in bacterial cells and populations. The DnaA-like, RepB, and RepABC replicons all carry type I partitioning systems, encoding Walker-type ATPases (Thomas, 1981) (Figure 2A). The vast majority of ECRs also have class II toxin-antitoxin (TA) systems, mainly encoding toxins of the VapC family (18 toxins), as well as toxins of the RelE/ParE (14), PhD/YefM (14) or parDE (8) families. None of the ECRs encode a complete type IV secretion system (T4SS), suggesting that these replicons are not self-transmissible. However, 16 of them contain genes encoding predicted relaxases of the Mob_C_ (14), Mob_Q_ (1), and Mob_HEN_ (1) families (Garcillán-Barcia et al., 2009), which are typical components of genetic modules, enabling plasmid mobilization for conjugative transfer (MOB).

Further analysis was conducted to verify whether the *P. yeei* genomes contain ECRs that meet the criteria for chromids (secondary chromosomes)—essential replicons of plasmid origin. This revealed that all the DnaA-like, RepB1 and RepB2 replicons can be considered chromids (Figure 1C), since they (i) carry plasmid-type REP modules, (ii) have a nucleotide sequence composition that is close to that of the chromosome (< 1% difference in GC content), and (iii) possess a set of core genes (in one copy in the genome) characteristic for the entire genus (Harrison et al., 2010). The genes carried by these chromids participate in several important metabolic pathways, e.g., encoding acetyl-CoA C-acyltransferase, and proteins likely to be involved in biosynthesis of the lipoyl cofactor, in oxidative metabolism, leucine metabolism, and electron transport via the respiratory chain (complex I).

The Rep proteins of the DnaA-like, RepB1 and RepB2 chromids display a high level of aa sequence identity within a given replicon group. These replicons are also more highly conserved in terms of their structure and genetic load (Supplementary Table S5). In contrast, the replication initiators of the RepABC, Rep_3 and HTH_36 families (and their entire replicons) show much greater sequence variability. Many of these replicons appear to be unique among *P. yeei* strains, suggesting their recent horizontal acquisition (Supplementary Table S5).

### 3.4 Comparative analysis of *P. yeei* genomes

Phylogenetic analysis showed that the two environmental isolates (CCUG 54214 and LM20) are situated on different branches of the *P. yeei* phylogenetic tree and are closely related to the clinical isolates. Strains LM20 and CCUG 46822 form a separate cluster, while the other five strains can be divided into two phylogenetic groups: (i) CCUG 54214 and CCUG 13493, and (ii) CCUG 17731, CCUG 32052 and CCUG 32054 (Figure 1A).

**Figure 3:**
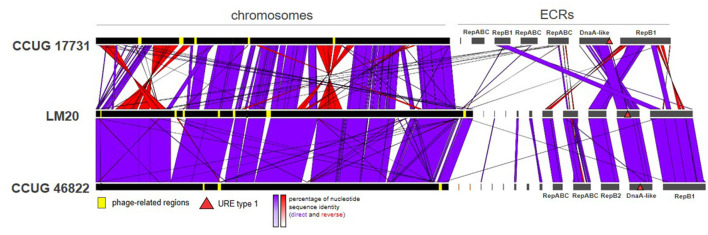
Comparative genomic analysis of three *P. yeei* strains, CCUG 17731, LM20, and CCUG 46822, illustrating the two chromosome structural variants identified in this species.

Comparative genomic analysis of the *P. yeei* strains (including clinical isolates CCUG 32053, FDAARGOS_643, FDAARGOS_252, and TT13) revealed a conserved genome structure, preserved ECR composition and synteny of gene arrangement in this species (Supplementary Table S5). There is a particularly high degree of synteny between the genomes of the strains LM20 and CCUG 46822 (Figure 3). The ECRs of these strains are nearly identical, although they display minor rearrangements (Supplementary Table S5).

Our analysis identified two chromosome structure variants (one found in strains CCUG 17731, CCUG 32052, CCUG 32053, CCUG 32054, CCUG 54214, FDAARGOS_643, TT13, and the other in CCUG 13493, CCUG 46822, LM20, FDAARGOS_252), which correlates with the phylogeny of the strains (Figure 3; Supplementary Table S5). The differences result mainly from inversions or translocations of fairly large DNA segments (Figure 3).

The DnaA-like replicons show a high degree of conservation, although with some rearrangements, e.g., translocation of DNA segments in strains CCUG 32053 and CCUG 13493, sometimes with inversion of the transferred DNA segment (Supplementary Table S5).

### 3.5 Genetic load of *P. yeei* genomes

**Figure 4:**
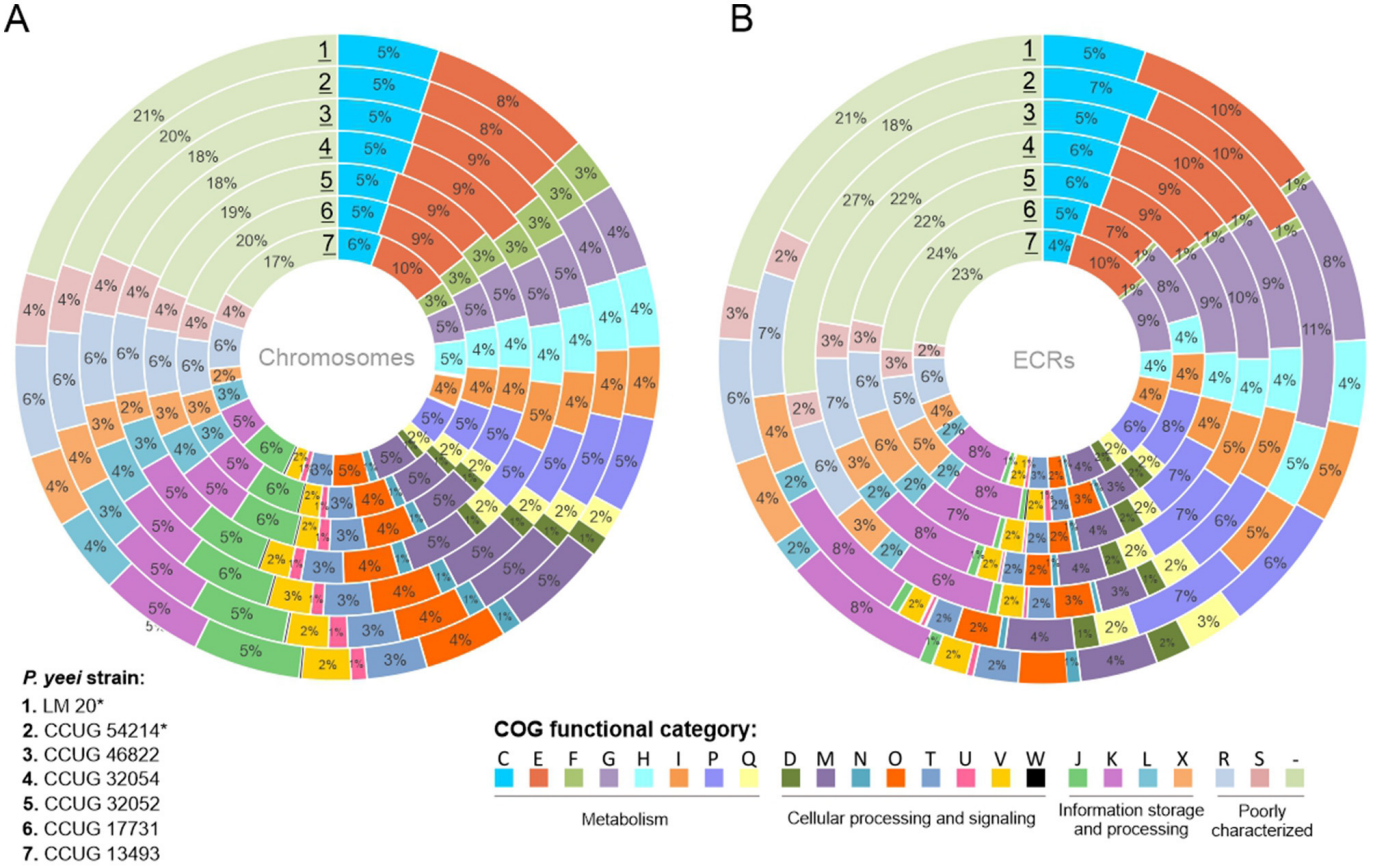
Distribution of genes encoding proteins belonging to different COG functional categories within **(A)** the chromosomes and **(B)** ECRs of the analyzed *P. yeei* strains (*environmental isolates). Each colored segment indicates the relative contribution of a functional category as a percentage of total COGs. Each ring represents a different strain of *P. yeei*. COG functional categories: C, energy production and conversion; E, amino acid transport and metabolism; F, nucleotide transport and metabolism; G, carbohydrate transport and metabolism; H, coenzyme transport and metabolism; I, lipid transport and metabolism; P, inorganic ion transport and metabolism; Q, secondary metabolite biosynthesis, transport and catabolism; D, cell cycle control, cell division, chromosome partitioning; M, cell wall/membrane/envelope biogenesis; N, cell motility; O, posttranslational modification, protein turnover, chaperones; T, signal transduction mechanisms; U, intracellular trafficking, secretion and vesicular transport; V, defense mechanisms; W, extracellular structures; Z, cytoskeleton; J, translation, ribosomal structure and biogenesis; K, transcription; L, replication, recombination and repair; X, mobilome, i.e., prophages, transposons; R, general function, prediction only; S, function unknown, COG not assigned.

Analysis of the proteome of *P. yeei* determined *in silico* permitted the assignment of individual proteins to functional COG groups. For all genomes a similar proportion of the encoded proteins belong to each category, indicating a conserved genome structure and limited variability within the species. Interestingly, significant similarity in the distribution of COG groups was found between the chromosomes and ECRs, suggesting that ECRs may contain a large amount of genetic information of chromosomal origin (Figure 4).

In all cases, a considerable number of genes are of unknown function (R and S categories) or could not be classified into any COG group: 27–31% for chromosomes and 27–32% for ECRs. The largest group of classified proteins comprises those related to amino acid transport and metabolism (category E; 9–10%), followed by carbohydrate metabolism (G; 5–6%), transcription (K; 6%), transport and metabolism of inorganic ions (P; 5–6%), energy acquisition processes (C; 5%), and the synthesis of cell wall, membrane, and envelope components, including capsules (M; 5%) (Supplementary Figure S2). In the case of ECRs, the largest fraction of classified proteins was assigned to categories E (7–10%), G (8–11%), P (6–8%), and K (6–8%) (Figure 4B). There are no significant differences between clinical and environmental isolates in the distribution of COG categories.

Transport-related genes constitute a dominant group of functional genes in the *P. yeei* genomes—from 530 (CCUG 32052) to over 600 CDSs (CCUG 17731). These genes encode various types of transporters: ABC, MSF (Major Facilitator Superfamily), P-type, RND (Resistance-Nodulation-Division), TRAP (Tripartite ATP-independent Periplasmic), and TTT (Tripartite Tricarboxylate Transporters) (Table 4; Supplementary Table S6). ECRs (mainly DnaA-like and RepB chromids) contain over 35% of the ABC transporter genes. Less common in ECRs are MFS, RND and TTT transporter genes. Only a few genes encoding proteins with similarity to P-type ATPase integral membrane transporters were found in the analyzed genomes.

**Table 4:** Distribution and characterization of transport-related genes in *P. yeei* genomes.

**Transporter type**	***P. yeei*** **clinical isolates**					**Environmental isolates**	
	**CCUG 13493**	**CCUG 17731**	**CCUG 32052**	**CCUG 32054**	**CCUG 46822**	**CCUG 54214**	**LM20**
**ABC No. of genes**	**366**	**390**	**370**	**384**	**341**	**342**		**342**
Chromosome (%)	219 (60%)	235 (60%)	241 (65%)	241 (63%)	225 (66%)	213 (62%)	226 (66%)
ECRs (%)	147 (40%)	155 (40%)	129 (35%)	143 (37%)	116 (34%)	129 (38%)	116 (34%)
**MSF**	**31**	**34**	**29**	**34**	**30**	**28**	**29**
Chromosome	22 (71%)	23 (68%)	23 (79%)	28 (82%)	22 (73%)	20 (71%)	21 (72%)
ECRs	9 (29%)	11 (32%)	6 (21%)	6 (18%)	8 (27%)	8 (29%)	8 (28%)
**P-type**	**4**	**7**	**4**	**5**	**3**	**8**	**6**
Chromosome	3 (75%)	4 (57%)	3 (75%)	4 (80%)	3 (100%)	8 (100%)	6 (100%)
ECRs	1 (25%)	3 (43%)	1 (25%)	1 (20%)	0	0	0
**RND**	**21**	**15**	**15**	**17**	**19**	**20**	**20**
Chromosome	10 (48%)	10 (67%)	10 (67%)	14 (82%)	11 (58%)	15 (75%)	11 (55%)
ECRs	11 (52%)	5 (33%)	5 (33%)	3 (18%)	9 (42%)	5 (25%)	9 (45%)
**TRAP**	**19**	**17**	**19**	**25**	**19**	**20**	**19**
Chromosome	11 (58%)	11 (65%)	13 (68%)	15 (60%)	10 (53%)	14 (70%)	10 (53%)
ECRs	8 (42%)	6 (35%)	6 (32%)	10 (40%)	9 (47%)	6 (30%)	9 (47%)
**TTT**	**16**	**15**	**13**	**16**	**19**	**27**	**16**
Chromosome	16 (100%)	13 (87%)	10 (77%)	13 (81%)	16 (84%)	19 (70%)	13 (81%)
ECRs	0	2 (13%)	3 (23%)	3 (19%)	3 (16%)	8 (30%)	3 (19%)
**Others/not defined**	**106**	**125**	**107**	**103**	**108**	**114**	**113**
Chromosome	84 (79%)	82 (66%)	81 (76%)	73 (71%)	82 (76%)	89 (78%)	87 (77%)
ECRs	22 (21%)	43 (34%)	26 (24%)	30 (29%)	26 (24%)	25 (22%)	26 (23%)
**Total**	**563**	**604**	**530**	**557**	**540**	**559**	**545**

The ECRs also carry numerous genes that may increase the adaptability of their host strains in the environment. All types of chromids and RepABC plasmids encode potential iron or carbohydrate transporters. Siderophore receptor genes were identified in all RepB2 plasmids, including the FhuADCB ferric hydroxamate transporter, which is essential for the uptake of Fe^3+^-aerobactin. All RepB1 replicons carry a *mauMGJCBDEAF* gene cluster involved in the utilization of methylamine (Baker et al., 1998). Organisms that utilize C1 compounds as their sole carbon and energy sources are described as methylotrophs and this trait is common in bacteria of the genus *Paracoccus* (Czarnecki and Bartosik, 2019). Methylotrophs play an important role in global carbon, nitrogen and sulfur cycling, and they have been successfully employed in the bioremediation of contaminated soils (Chistoserdova, 2011).

The RepB1 replicons of three phylogenetically closely related *P. yeei* strains (CCUG 17731, CCUG 32052, CCUG 32054) (Figure 1A) contain a putative operon (*phnCDEFGHIJKLMNOP*) encoding carbon-phosphorus lyase, which permits the utilization of phosphorus from a wide range of stable phosphonate compounds containing a C-P bond (Makino et al., 1991; Horsman and Zechel, 2017; Amstrup et al., 2023). In numerous environments, inorganic phosphate, a vital nutrient, is available in very limited amounts, compelling microorganisms to rely on alternative phosphorus sources for survival (Podzelinska et al., 2009). Therefore, the ability to uptake and breakdown phosphonates would be advantageous.

Enzymes catalyzing rhamnose biosynthesis (RfbABCD) are encoded by all RepB2 replicons (CCUG 32052 has an additional copy of the *rfbABCD* operon in Rep_3 plasmid pYEE32052P3). Rhamnose is present in the O-antigens of many gram-negative bacteria, forming part of the lipopolysaccharide (LPS). This deoxy-hexose sugar is also present in capsular polysaccharides, covalently bound to the cell wall, and in exopolysaccharides that are loosely associated with the cell wall (Marolda and Valvano, 1995). The *P. yeei* strains analyzed in this study display characteristic mucoid growth, probably related to the presence of a polysaccharide capsule (data not shown).

The ECRs also carry genes for proteins involved in (i) the cobalamin (vitamin B12) biosynthetic pathway (*cobAWNGHIJKLMBF*; DnaA-like chromids), (ii) ectoine biosynthesis (*ehuABCD*; RepB1 chromids), (iii) propionate catabolism (*mmgE/prpD*; several RepB1, RepB2, and RepABC replicons), (iv) disulfide bond formation (*dsbA, dsbB*, and *dsbE*), and (v) resistance to arsenic compounds (permease Arc3, arsenate reductase ArsC, flavoprotein ArsH, repressor ArsR, and transporter ArsJ). Notably, all RepB1 chromids carry a complete conserved set of *crt* genes (*crtXYIBZE-idi*) for carotenoid synthesis (Maj et al., 2013). However, colonies of the strains with these chromids lack the characteristic color associated with carotenoid pigment production and these compounds could not be detected by HPLC analysis (data not shown).

In summary, the DnaA-like, RepB1 and RepB2 chromids and the RepABC plasmids carry the largest number of genes of adaptive potential. The genetic load of smaller replicons (HTH_36 or Rep_3) consists mainly of genes of unknown function. The distribution of selected genes in individual ECRs is presented in Supplementary Table S3.

### 3.6 Transposable elements

**Figure 5:**
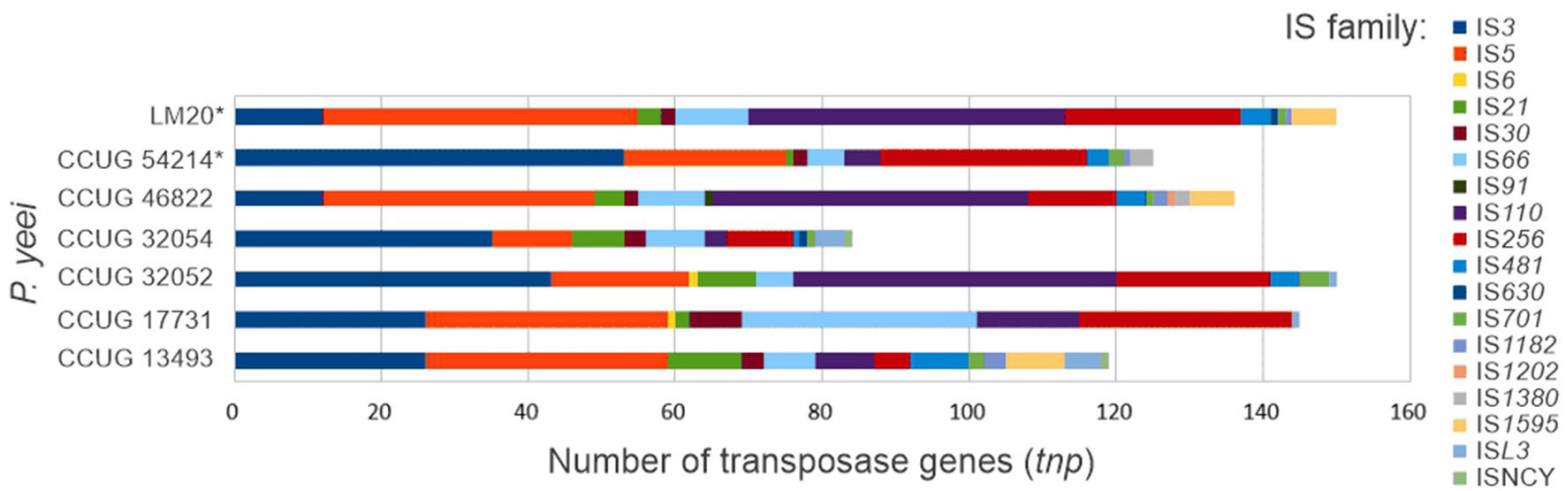
Distribution of insertion sequences in *P. yeei* genomes (*environmental isolates).

A search for transposable elements (TEs) in the *P. yeei* genomes revealed the presence of over 900 *tnp* genes (complete or partial) encoding transposases from 18 families of insertion sequences (ISs): IS*3*, IS*5*, IS*6*, IS*21*, IS*30*, IS*66*, IS*91*, IS*110*, IS*256*, IS*481*, IS*630*, IS*701*, IS*1182*, IS*1202*, IS*1380*, IS*1595*, IS*L3*, and ISNCY (Figure 5; Supplementary Table S7). The number of *tnp* genes in individual genomes ranges from 84 (CCUG 32054) to 150 (CCUG 32052 and LM20). The dominant elements are representatives of the IS*3*, IS*5*, IS*110*, and IS*256* families. Several IS families were identified in only one or a few isolates, e.g., IS*6—*CCUG 17731 and CCUG 32052, IS*91*—CCUG 46822, IS*630*—CCUG 32054 and LM20, IS*1202*—CCUG 46822, IS*1380*—CCUG 46822, IS*1595*—CCUG 13493, CCUG 46822 and LM20, and ISNCY—CCUG 13493 and CCUG 32054 (Supplementary Table S7).

Numerous ISs are localized within ECRs. Surprisingly, most of the largest ECRs (DnaA-like and RepB chromids), except for those from CCUG 46822 and LM20, contain relatively few ISs, indicating a more conserved replicon structure and the presence of essential genes. The replicons richest in these elements are RepABC plasmids, which in most cases carry almost 50% of the *tnp* genes of a given strain (Supplementary Table S7).

In the chromosomes and ECRs of strains CCUG 46822 and LM20 several repetitive IS arrays were identified with a structure resembling composite transposon (Supplementary Table S7). These DNA regions are flanked by divergently oriented complete isoforms of IS*Pye46* (1361 bp; IS*110* family) and contained within them are *tnp* genes of the IS*256* family (related to IS*Pye43*) and/or the IS*5* family (related to IS*Pye12*). Each copy of IS*Pye46* is flanked by identical DRs (5′-TG-3′), so it is not clear whether these IS*110*-IS*5*/IS*256*-IS*110* arrays constitute functional TEs or are the result of preferential transposition of IS*Pye46* into ISs representing the IS*256* or IS*5* families. Interestingly, IS*Pye46* is dominant in the analyzed *P. yeei* genomes (47 copies in total); however, most of the copies are present in strains CCUG 46822 (19 copies) and LM20 (24 copies) (Figure 5; Supplementary Table S7). No related IS*110*-IS*5*/IS*256*-IS*110* arrays were identified in the genomes of other bacteria by BLASTn analysis.

**Figure 6:**
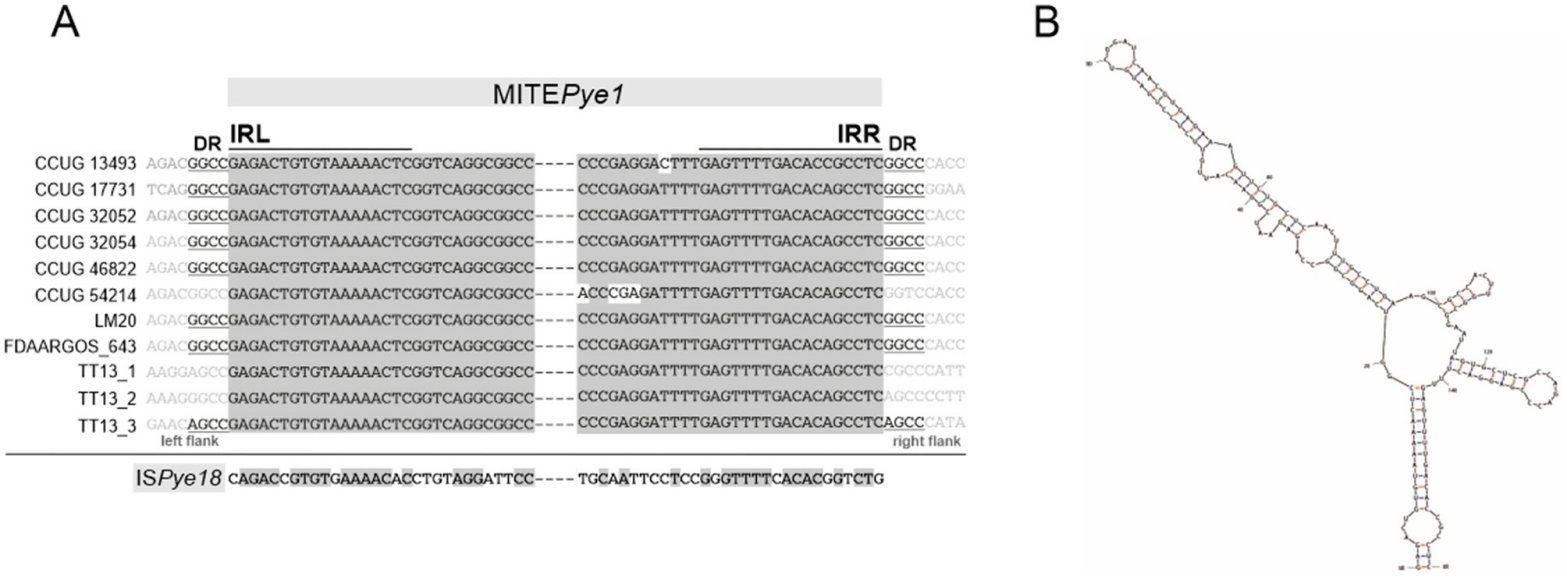
Non-autonomous transposable element MITE*Pye1* identified in *P. yeei* strains. **(A)** Alignment of terminal inverted repeat nucleotide sequences (IRL, left IR; IRR, right IR) of complete MITE*Pye1* elements identified in *P. yeei* genomes and IS*Pye18*. Identical residues are indicated by gray shading. Nucleotide sequences of direct repeats (DRs) generated by MITE*Pye1* elements during transposition are indicated by underlining. **(B)** RNA secondary structures predicted by *in silico* folding using Mfold software for MITE*Pye1*. The minimum folding energy (ΔG −47.93) of the predicted secondary structures was calculated by Mfold.

Interestingly, a 159-bp-long sequence element was identified in most chromosomes of the analyzed *P. yeei* strains (Supplementary Table S7). These elements show high sequence similarity (96–100% identity), lack any open reading frames, occur in intergenic regions at different locations, and are flanked by short (4 bp) direct repeats (DRs), suggesting their acquisition through transposition (Supplementary Table S7). Additionally, they contain inverted repeats at both termini (TIRs; IRL and IRR) (18 bp) with significant similarity to the TIRs of insertion sequences of the IS*1182* family, including IS*Pye18*, previously identified in the CCUG 32053 genome (Lasek et al., 2018) (Figure 6A). Analysis of the distribution of IS*Pye18* in *P. yeei* genomes revealed the presence of intact isoforms of this element in strains FDAARGOS_252 (1 copy; plasmid 4) and CCUG 32053 (1 copy; plasmid pYEE3). However, related ISs from the IS*1182* family were also identified in the genomes of CCUG 13493, CCUG 46822, CCUG 54214, and LM20 (Supplementary Figure S3).

Considering (i) the potential mobility of these DNA regions, (ii) their small size, (iii) the presence of TIRs, (iv) DRs, and (v) the absence of a transposase gene, it seems likely that they constitute a novel group of MITE-type non-autonomous TEs (Delihas, 2011), that are most probably mobilized for transposition by ISs of the IS*1182* family. This element was named MITE*Pye1* (ISfinder).

The activity of TEs of *P. yeei* was investigated *in vivo* using trap plasmids that allow positive selection of transposition events. Two trap plasmids were used, pMEC1 and pMAT1, containing the *cI*-*tetA* and *sacB* selection cassettes, respectively. Transposition of TEs into these cassettes resulted in the appearance of new phenotypes—the cells carrying a mutated plasmid become resistant to tetracycline (pMEC1) or to sucrose (pMAT1) (see Section 2 for details).

Despite the presence of a very large number of transposase genes in the *P. yeei* genomes, use of the entrapment vectors led to the identification of only six distinct elements: (i) three ISs previously identified *in silico* in the CCUG 32053 genome—IS*Pye2* (IS*5* family, IS*5* group), IS*Pye38* (IS*5* family) and IS*Pye41* (IS*5* family, IS*903* group), (ii) an isoform of IS*1247* of *Xanthobacter autotrophicus* (IS*1380* family), and (iii) two novel elements, designated IS*Pye79* and IS*Pye80*, representing the IS*5* and IS*427* groups within the IS*5* family, respectively.

The distribution of every defined IS of *P. yeei* (ISfinder and this study) across all *P. yeei* genomes is shown in Supplementary Figure S3. The most abundant elements are (i) IS*Pye46*−47 copies in 5 strains (most copies present in CCUG 46822 and LM20), (ii) IS*Pye53*−45 copies in seven strains, and (iii) IS*Pye41*−30 copies in six strains (most copies present in CCUG 17731 and CCUG 32053). Many of the ISs are present in only 1 copy, e.g., (i) IS*Pye13* (chromosome FDAARGOS_252), (ii) IS*Pye63* (chromosome of CCUG 32052), and (iii) IS*1247*a (chromosome of CCUG 46822).

### 3.7 *P. yeei* strain- and species-specific genes

The core genomes of *Paracoccus* spp. and of *P. yeei* were defined based on the complete genomic sequences of *P. aminophilus* JCM 7686 (Dziewit et al., 2014), *P. aminovorans* JCM 7685 (Czarnecki et al., 2017), *P. contaminans* RKI16-01929T (Aurass et al., 2017), *P. denitrificans* PD1222, plus eleven *P. yeei* strains. Comparative genomic analysis was used to define (i) the core genome of bacteria of the genus *Paracoccus* spp., (ii) genes specific to *P. yeei* (not found in other *Paracoccus* spp. with complete genomes), and (iii) genes that are unique to individual *P. yeei* strains (singletons). The collected data, also showing the distribution of these genes in the individual replicons of each *P. yeei* strain, are presented in Supplementary Table S8.

This analysis revealed that the core genome of *Paracoccus* spp. consists of 1,647 protein-encoding genes, of which 116 are located within ECRs of *P. yeei*. The total number of *P. yeei*-specific genes is 250 and the number of singletons ranges from 138 (CCUG 46822) to 332 (FDAARGOS 252) (Supplementary Table S8). In some strains, the majority of singletons occur in the chromosome, while in others (CCUG 13493, CCUG 17731) they are predominantly in ECRs. The vast majority of singletons encode proteins of unknown function, although a predicted role in adaptation could be assigned to some genes from the *P. yeei* environmental isolates. The chromosome of LM20 encodes (i) the membrane protein YeeE, which mediates the uptake of thiosulfate as an inorganic sulfur source for cysteine synthesis (Tanaka et al., 2020), (ii) mercury transporter Mer, and (iii) the sensor kinase KdpD, which senses potassium levels, and the *kdpABC* operon, which encodes a high-affinity potassium uptake system crucial for bacterial survival in low potassium environments (Laermann et al., 2013; Ali et al., 2017). In the CCUG 54214 genome, many transport-related genes (ABC, RND, TTT types) were distinguished among the singletons, as well as genes for toxin-antitoxin systems (e.g., HipA-, PrlF-, Yha- family), similarly to clinical strains.

The largest group of *P. yeei*-specific genes (found in chromosomes and DnaA-like, RepB1, RepB2 and RepABC replicons) encode transposases from different IS families. The second largest group of genes encodes transporters, mostly of the ABC type, which are located in chromosomes and in DnaA-like and RepB1 chromids. Other abundant genes, found mainly in RepB1 chromids, encode transcription regulators of the LuxR, CopG, TetR/AcrR, MarR, LacI, AraC, TetR, DeoR/GlpR, and NikR families (Supplementary Table S8).

Examples of proteins encoded by other *P. yeei*-specific genes include a urease enzyme, components of the trehalose, rhamnose, and cobalamin biosynthetic pathways, chaperone proteins, and factors possibly involved in the cell's response to stress conditions (Supplementary Table S8).

### 3.8 Putative virulence determinants

The proteomes of a number of *Paracoccus* strains were analyzed in order to identify potential virulence factors: (i) the seven *P. yeei* strains described in this study, (ii) other *P. yeei* strains whose complete genomic sequences are available (CCUG 32053, TT13, FDAARGOS_262, FDAARGOS_643), as well as (iii) several environmental isolates of the genus *Paracoccus*, including the type strain *P. denitrificans* PD1222. These protein sets were compared against databases containing bacterial virulence factors and antibiotic resistance genes (see Section 2 for details). Relatively few of the identified determinants were unique to *P. yeei* (all clinical isolates). The pool of identified genes includes those encoding six types of protein, that were previously mentioned by Lasek et al. (2018), i.e., (i) methionine sulfoxide reductase (*msrA1, msrA2, msrB*), (ii) diguanylate cyclase, (iii) superoxide dismutase, (iv) sugar transferase, (v) type IV secretion system components, and (vi) urease (Supplementary Tables S3, S9).

The chromosomally-located *msr* genes of CCUG 32052 and CCUG 46822 encode predicted proteins sharing 61–100% aa sequence similarity, while the homologous proteins encoded by other *P. yeei* strains are less well-conserved (38–55% aa identity). Msr proteins participate in the detoxification of reactive oxygen intermediates (both prokaryotic and eukaryotic cells lacking Msr are sensitive to oxidative stress) (Denkel et al., 2011). Moreover, MsrA has been shown to be critical for the survival of *Erwinia chrysanthemi* (El Hassouni et al., 1999), *Mycoplasma genitalium* (Das et al., 2012), and *Helicobacter pylori* (Alamuri and Maier, 2006) in their infected hosts. Genes encoding components of a putative type IV secretion system (T4SS) were identified only in CCUG 17731 (30–50% aa identity) and in CCUG 13493 (25–50% aa identity)—within RepABC plasmids in both cases. However, these were not complete modules, unlike the one previously identified in CCUG 32053 (Lasek et al., 2018).

The remaining virulence factors, present in all analyzed genomes, include the gene encoding diguanylate cyclase, an enzyme synthesizing cyclic diguanylate (c-di-GMP)—an important bacterial second messenger. This signaling molecule is involved in the regulation of a number of complex physiological processes, including biofilm formation and motility, which affect the pathogenesis of many bacteria (Tamayo et al., 2007).

Genes encoding several other putative virulence factors were identified within ECRs, although they were not unique to *P. yeei*. These include proteins participating in the following: (i) numerous ABC and RND transport systems (DnaA-like, RepB, RepABC replicons), (ii) siderophore synthesis (RepB2 replicons, chromosomes), (iii) polysaccharide capsule production (RepB2 replicons), and (iv) trehalose synthesis (DnaA-like type replicons).

All *P. yeei* strains contain a set of genes (URE) involved in the synthesis of the metalloenzyme urease, which hydrolyzes urea to ammonia and CO_2_. These genes are located within essential replicons—DnaA-like chromids. They display synteny and a high degree of nucleotide sequence identity (98%), indicating their biological importance. The role of urease in bacterial pathogenesis is well-documented (e.g., Mobley, 1996; Dupuy et al., 1997; Burne and Chen, 2000; Berutti et al., 2014; Rutherford, 2014; Graham and Miftahussurur, 2018; Zhou et al., 2019; Minami et al., 2021).

### 3.9 URE gene clusters

**Figure 7:**
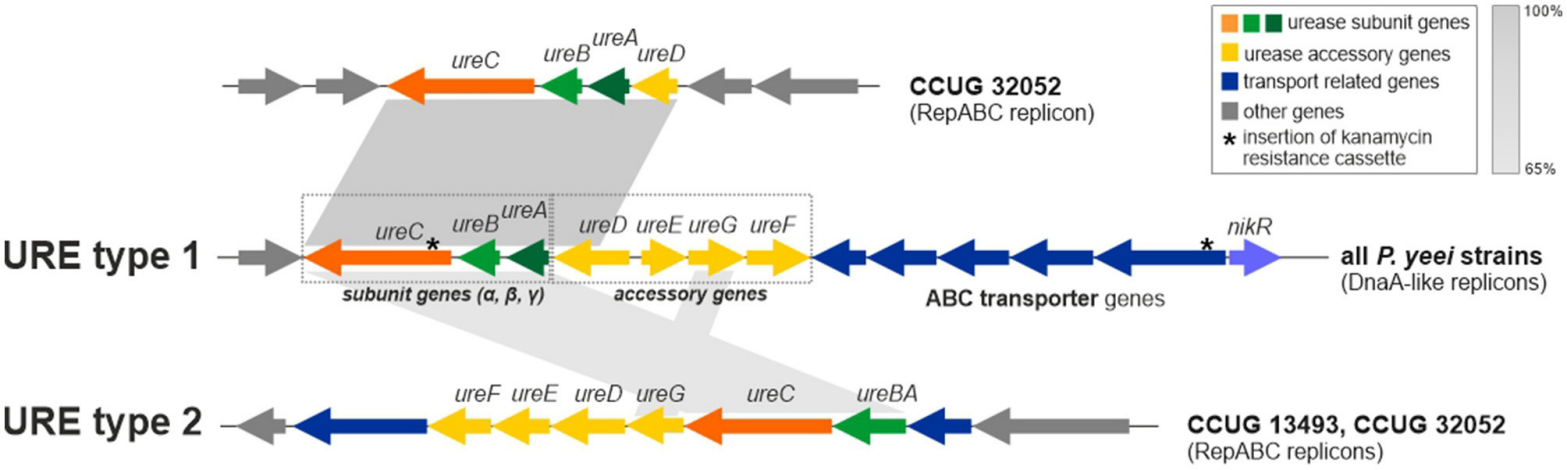
Genetic organization of urease gene clusters (URE) located in *P. yeei* ECRs—URE type 1 (present in all DnaA-like chromids) and URE type 2 (present in two RepABC plasmids of *P. yeei* CCUG 13493 and CCUG 32052). Shaded areas connect homologous DNA regions.

Besides genes for the main urease subunits (UreA—γ subunit, UreB—β subunit, and UreC—α subunit) and accessory proteins (*ureD, ureE, ureF*, and *ureG*), the URE gene cluster also contains the *nikR* gene (encoding a predicted nickel-dependent transcription factor) and numerous ABC transporter genes, likely to be involved in the uptake of Ni^2+^ (an essential cofactor for urease; Rutherford, 2014). It is probable that NikR regulates the expression of the transporter genes (Figure 7). Interestingly, in *P. yeei* strain CCUG 32052, part of the URE gene cluster, containing the *ureABC* and *ureD* genes, has been duplicated and transferred to another ECR (RepABC; pYEE32052P4) (99% nucleotide sequence identity to the corresponding URE genes) (Figure 7).

The URE gene cluster of DnaA-like replicons (designated URE type 1) (Figure 7) is unique to and characteristic of *P. yeei* strains. However, in two strains (CCUG 13493 and CCUG 32052) another set of *ure* genes (URE type 2; Figure 7), possibly associated with urease synthesis, was identified. These genes are present in RepABC plasmids in both strains (pYEE13493P4 and pYEE32052P5, respectively) and their genetic organization is different from that of URE type 1. In the URE type 2 cluster, the γ and β urease subunits are encoded by a single fusion gene *ureBC*, the order and transcriptional orientation of the accessory genes *ureFEDG* is different, there are fewer transporter genes and the *nikR* gene is absent (Figure 7).

URE type 2 represents a novel type of ureolytic gene cluster which has a different evolutionary origin from URE type 1. The two clusters share some sequence similarity between the genes encoding the main urease subunits, but no similarity was found in case of the accessory genes and their arrangement is different.

The ureolytic activity of three *P. yeei* strains (CCUG 32053, CCUG 32052—containing the duplicated part of URE type 1, and CCUG 32052—also carrying URE type 2; Figure 7) was examined using Christensen's differential medium without (control) and with the addition of urea (1%) (see Section 2 for details). All the strains displayed ureolytic activity, which was induced by the presence of urea in the medium.

To unequivocally confirm the role of the type 1 URE gene cluster in the observed ureolytic activity, mutations were introduced by insertion of a kanamycin resistance cassette into the *ureC* (encoding the α subunit of urease) or *nikR* genes of CCUG 32053 and CCUG 13493. These mutations completely abolished ureolytic activity in these strains, indicating the importance of both *ureC* and *nikR* in determining this phenotype. The lack of ureolytic activity in mutated strain CCUG 13493, which also contains URE type 2, indicates that this second cluster does not produce an active urease under the tested conditions and its genes are unable to complement mutations introduced in URE type 1.

## 4 Discussion

These comprehensive comparative genomic analyses of a pool of strains of the opportunistic bacterium *P. yeei*, have provided valuable information on (i) the structural diversity of genomes within this species, (ii) the components of the mobilome, their impact on genome structure and properties, and (iii) the genetic information determining the opportunistic properties of this species. Importantly, the analyzed strains originated from different environments (clinical and natural) and were isolated at different times. Therefore, the collected data better reflects the extent of strain variability within this species, and also permits the identification of genes conserved during evolution, including those involved in the process of pathogenesis (Table 1).

The physiological and phenotypic properties of the analyzed strains were found to be very similar. High levels of similarity were also evident when the structure and genetic content of the genomes of clinical and environmental isolates were compared. The relatedness of these strains is reflected in the topology of the *P. yeei* phylogenetic tree (Figure 1A).

All strains contain numerous ECRs of varying sizes, ranging from 4 to 485 kbp. It should be noted that strain CCUG 46822 carries the largest number of ECRs (13) among *Paracoccus* spp. strains analyzed so far. The main ECRs are large replicons representing the DnaA-like, RepB (RepB1 and RepB2 subgroups) and RepABC families, which is consistent with our previous observations (Lasek et al., 2018) (Figures 1B, C). All of the DnaA-like, RepB1 and RepB2 replicons (which meet the criteria of chromids) contain numerous adaptive genes (facilitating survival in challenging environmental conditions) plus a set of conserved genes of the core *Paracoccus* spp. genome. However, the genetic load of these replicons is not equal. DnaA-like replicons can be considered major chromids due to (i) the presence of the largest number of core genes, (ii) the relatively low variability in their structure and size (especially in relation to DnaA-like replicons of other *Paracoccus* species) (Figure 3; Supplementary Table S5), and (iii) the presence of the URE gene cluster, which is chromosomally located in other pathogenic bacteria.

DnaA-like chromids are characteristic for *Paracoccus* spp. and play an important role in the biology of these bacteria. Related essential replicons were previously identified and analyzed e.g., in *P. denitrificans* PD1222, *P. aminophilus* JCM 7686 and *P. aminovorans* JCM 7685 (Dziewit et al., 2014; Czarnecki et al., 2017). The removal of DnaA-like and RepB chromids from *P. aminophilus* cells was attempted (Dziewit et al., 2014), but this was only successful in the latter case. The strain lacking RepB replicons grew much more slowly on complete medium and was unable to grow at all on minimal media (regardless of the type of carbon source). Based on these observations, two classes of chromids were proposed: obligatorily essential primary chromids (e.g., DnaA-like) and facultatively essential secondary chromids (e.g., RepB).

It should be noted that DnaA-like chromids have yet to be identified in other taxonomic groups of bacteria, which is consistent with the assertion of Harrison et al. (2010) that particular chromid types are characteristic for a specific taxonomic group of bacteria (mainly genus). This claim seems reasonable given that these types of replicons are likely to have arisen from plasmids (potentially with a narrow host range) specific to a particular group of bacteria. DnaA-like replicons therefore have some aptitude to generate chromids. However, it has yet to be explained why, for example, RepABC replicons, which are the main chromids of e.g.. *Rhizobium* spp. and *Agrobacterium* spp. (Landeta et al., 2011; diCenzo et al., 2013; Döhlemann et al., 2017), despite also being common in *Paracoccus* spp., do not play the same role in these bacteria.

The flexible genome of *P. yeei* also comprises transposable elements. A previous study examining strain CCUG 32053 highlighted an unusual diversity of insertion sequences, not seen in other *Paracoccus* spp. strains (Lasek et al., 2018). This analysis resulted in the examination of the prevalence of 80 ISs (IS*Pye*1–IS*Pye*80) sequences in the genomes of *P. yeei*, that had not been previously characterized in the transposable mobilome of *Paracoccus* spp. (Dziewit et al., 2012). In this study, we searched for putative TEs in all available complete genomic sequences of *P. yeei*. The results of these analyses showed (i) the widespread presence of ISs in this species (although there is great variability in their number in individual strains), (ii) the predominance of ISs belonging to the IS*5*, IS*3*, IS*110*, and IS*256* families (Figure 5; Supplementary Figure S3), and the absence of (iii) *P. yeei*-specific elements conserved in all genomes or (iv) transposons that could be linked with potential determinants of pathogenesis. A more global comparative analysis using data collected in the ISfinder database showed that the overall distribution pattern of IS families in the *P. yeei* genomes corresponds to their distribution across the genus *Paracoccus*. This also highlighted the ubiquity and diversity of these elements in *Paracoccus* spp. in comparison to other genera of *Alphaproteobacteria* (Supplementary Figure S4).

The presence of multiple copies of particular ISs in *P. yeei* genomes may promote homologous recombination, resulting in genomic structural rearrangements. It is likely that such events led to the generation of the two chromosome structural variants identified in this study (Figure 3). Notably, the large number of TEs predicted *in silico* did not correlate with the number of active elements identified by the application of trap plasmids. Only a few elements that were the most dynamic in the process of transposition were captured. The low activity of TEs may be due to the presence of multiple regulatory systems keeping this process in check to reduce the likelihood of lethality caused by insertional inactivation of host housekeeping genes (Lipszyc et al., 2022).

Analysis of the *P. yeei* genomes also identified a novel group of non-autonomous TEs (MITE-type; MITE*Pye1*). These elements contain TIRs and are bordered by DRs, so resemble functional TEs in this respect (Figure 6A). Their TIRs share significant sequence similarity with terminal sequences of IS*1182*-family elements, which suggests that they originate from defective ISs and their transposition may be trans-activated by compatible transposases. In the chromosomes, these MITE-type elements occur in low numbers (1–3) and are preferentially located within intergenic regions (Supplementary Table S7). Mfold analysis revealed that these elements are able to fold into long stem-loop structures at the RNA level (Figure 6B). Therefore, their co-transcription with upstream genes, may influence the conformation and stability of the resulting transcripts. The altered expression may result in various phenotypes depending on the specific gene function (Szuplewska and Bartosik, 2009; Dziewit et al., 2012; Szuplewska et al., 2014).

Particular attention was given to the identification of genes responsible for the opportunistic phenotype of *P. yeei*. Collections of *P. yeei* species-specific genes were searched for potential virulence factors. A noteworthy finding of this analysis was the very large number of transporter genes, which is a unique feature among *Paracoccus* spp. Although it is difficult to directly link their presence to the ability of these bacteria to cause opportunistic infections, they could potentially play an important role in pathogenesis.

The URE gene clusters of *P. yeei* identified in the screen for potential pathogenicity determinants were examined in more detail. These clusters are involved in the synthesis of urease, an enzyme that acts as a virulence factor in many pathogenic bacteria, including *Staphylococcus* spp. (90% of methicillin-resistant *S. aureus* display ureolytic activity), *Helicobacter pylori, Mycobacterium tuberculosis, Mycobacterium bovis*, anaerobic *Clostridium perfringens* and *Vibrio parahaemolyticus* (Dupuy et al., 1997; Berutti et al., 2014; Graham and Miftahussurur, 2018; Zhou et al., 2019; Minami et al., 2021). In the case of *H. pylori*, urease is one of the major virulence factors, since strains unable to produce this enzyme cannot colonize the gastric mucosa. The ammonia released by urease action affects the tissue, causing damage to epithelial cells. Urease activity also enables pathogens to survive and proliferate in macrophages (Fu et al., 2018). This enzyme elicits a response from the human immune system by stimulating the production of antibodies (Konieczna et al., 2012).

Two diverse URE gene clusters were identified in the *P. yeei* genomes: type 1—species specific, and type 2—present only in two RepABC plasmids. We showed that URE type 1 determined the urease activity, which was induced by the presence of urea in the growth medium. Activity of the URE type 2 cluster could not be demonstrated. It may be speculated that these URE module variants require different substrates/activators to initiate the synthesis of urease. Nevertheless, it is highly probable that the presence of additional URE gene clusters may enhance the ureolytic capacity of the bacteria, which highlights the biological importance of this phenotype. It is important to note that urea is present in large quantities in the human body, mainly in the kidneys, but is also found in the stomach, blood serum, sweat and milk (Fu et al., 2018; Graham and Miftahussurur, 2018; Schimmel et al., 2021).

Mutations introduced into the *ureC* (encoding a subunit of urease) or *nikR* (encoding a putative regulator of nickel transporter gene expression) genes of URE type 1 completely abolished urease activity. This effect was previously observed in analogous mutants of *H. pylori*. One of the main phenotypes of an *H. pylori nikR* mutant was the absence of nickel-responsive induction of urease expression (Van Vliet et al., 2002). Further studies showed that NikR binds to the *ureA* promoter (in a nickel-dependent manner), which results in nickel-induced transcription and expression of a ureolytic phenotype (Ernst et al., 2005). We hypothesize that the *P. yeei ure* genes may be regulated in a similar way.

As the conserved URE type 1 gene cluster of *P. yeei* is unique among *Paracoccus* spp., it is highly probable that urease plays an important role in the pathogenesis of this species. The verification of this hypothesis is an immediate goal of our future studies.

## Data Availability

The datasets presented in this study can be found in online repositories. The names of the repository/repositories and accession number(s) can be found in the article/Supplementary material.
