# Supplementary material for: Genome-wide comparative analysis of clinical and environmental strains of the opportunistic pathogen Paracoccus yeei (Alphaproteobacteria)
Source: Front Microbiol. 2024 Nov 6;15:1483110. doi: 10.3389/fmicb.2024.1483110 (PMC11578231; doi:10.3389/fmicb.2024.1483110)
Supplement: Supplementary file 1 [file Data_Sheet_1.PDF]

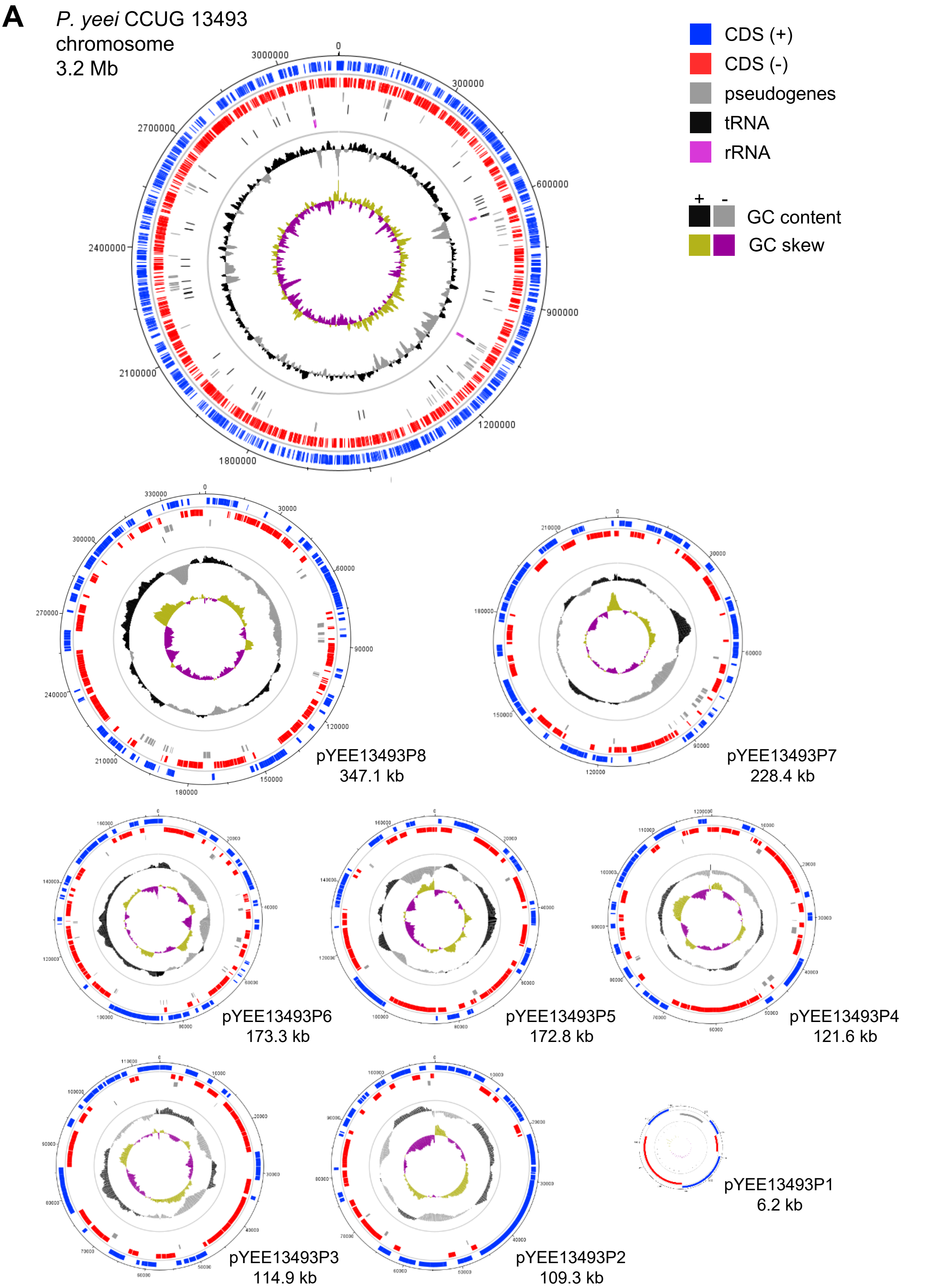

Figure S1. Genome structure of *Paracoccus yeei* strains. (**A**) CCUG 13493; (**B**) CCUG 17731; (**C**) CCUG 32052; (**D**) CCUG 32054; (**E**) CCUG 46822; (**F**) CCUG 54214; (**G**) LM20. The tracks from the outside represent: (1) CDS forward strand (blue); (2) CDS – reverse strand (red); (3) pseudogenes (grey); (4) tRNA (black); (5) rRNA (purple); (6) %GC content; (7) GC skew. Circles are drawn not in scale. Figure generated using DNAPlotter (Carver et al., 2009).

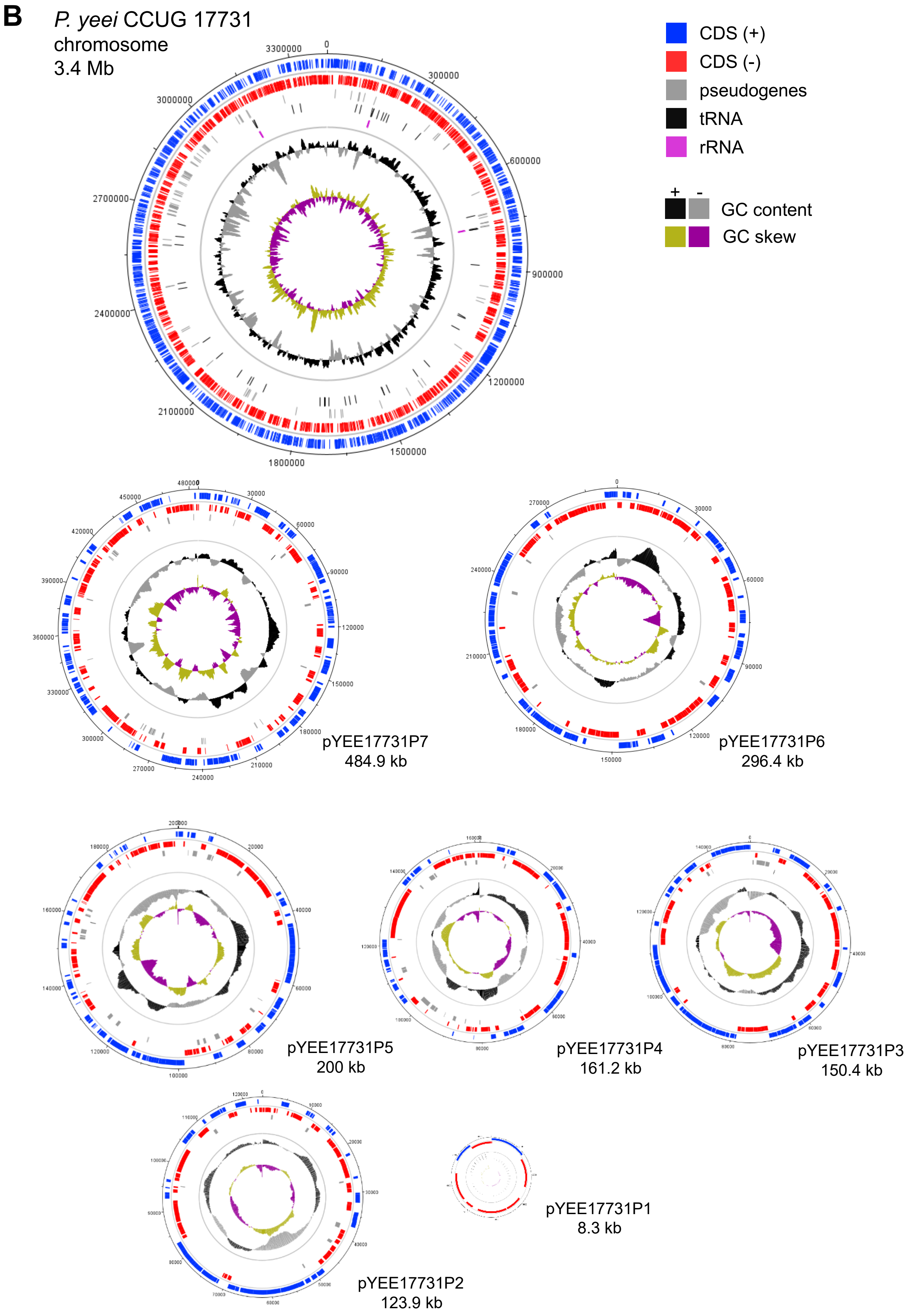
B
P. yeei CCUG 17731
chromosome
3.4 Mb
CDS (+)
CDS (-)
pseudogenes
tRNA
rRNA
+ -
GC content
GC skew
0
300000
600000
900000
1200000
1500000
1800000
2100000
2400000
2700000
3000000
3300000
pYEE17731P7
484.9 kb
pYEE17731P6
296.4 kb
pYEE17731P5
200 kb
pYEE17731P4
161.2 kb
pYEE17731P3
150.4 kb
pYEE17731P2
123.9 kb
pYEE17731P1
8.3 kb

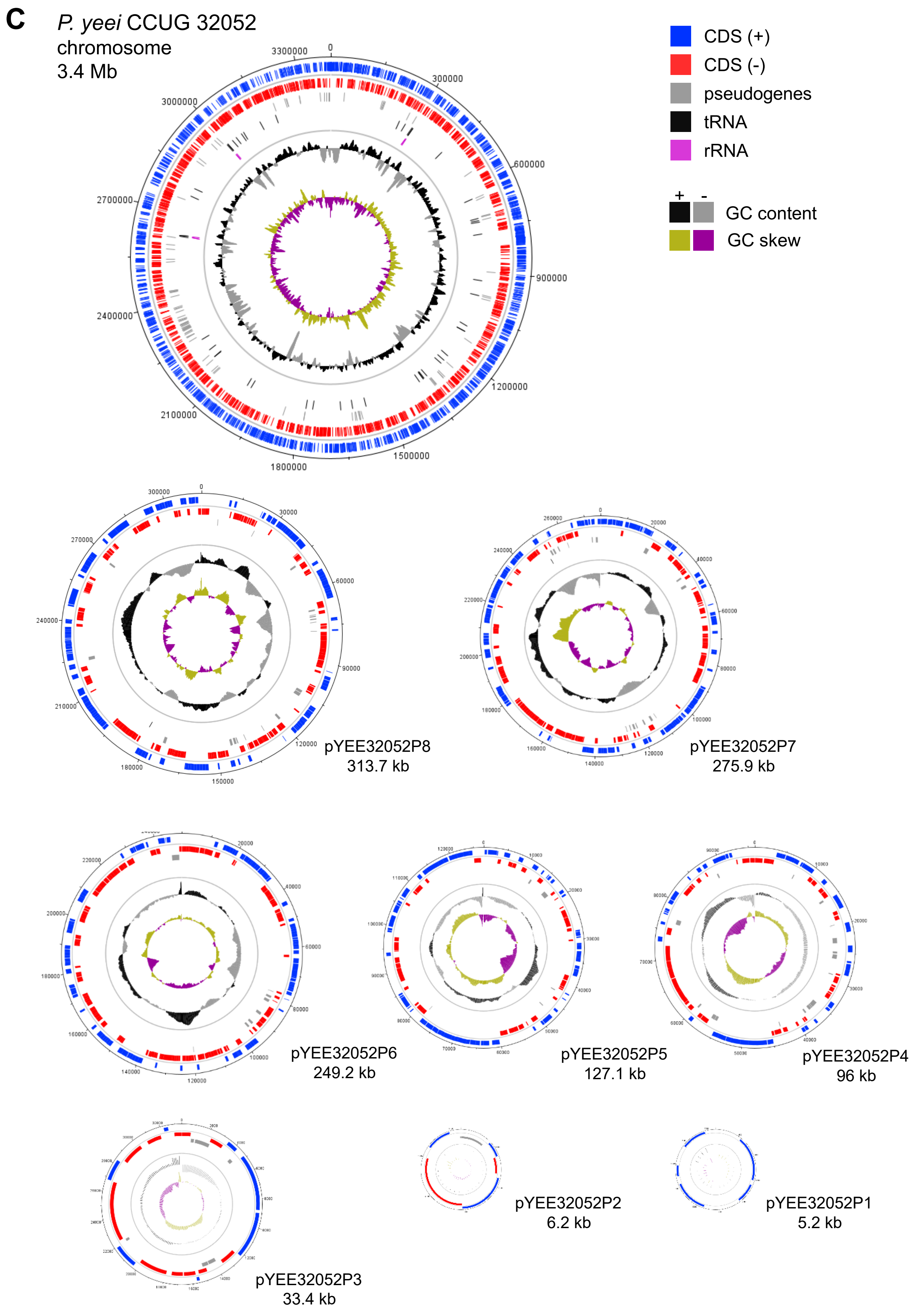
C
P. yeei CCUG 32052
chromosome
3.4 Mb
0
300000
600000
900000
1200000
1500000
1800000
2100000
2400000
2700000
3000000
3300000
CDS (+)
CDS (-)
pseudogenes
tRNA
rRNA
+ -
GC content
GC skew
pYEE32052P8
313.7 kb
pYEE32052P7
275.9 kb
pYEE32052P6
249.2 kb
pYEE32052P5
127.1 kb
pYEE32052P4
96 kb
pYEE32052P3
33.4 kb
pYEE32052P2
6.2 kb
pYEE32052P1
5.2 kb

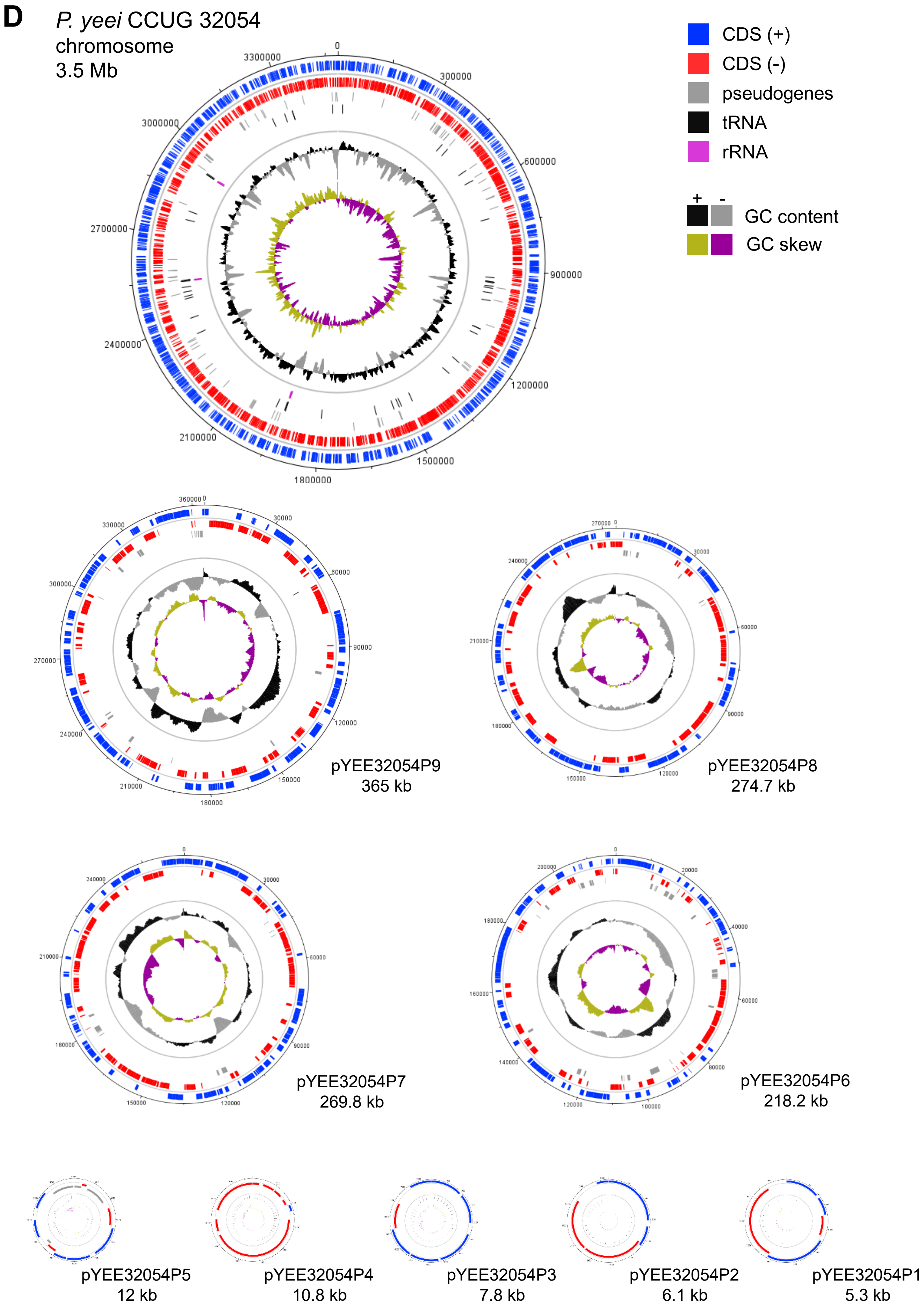
D
P. yeei CCUG 32054
chromosome
3.5 Mb
0
300000
600000
900000
1200000
1500000
1800000
2100000
2400000
2700000
3000000
3300000
CDS (+)
CDS (-)
pseudogenes
tRNA
rRNA
+
-
GC content
GC skew
360000 0
30000
60000
90000
120000
150000
180000
210000
240000
270000
300000
330000
pYEE32054P9
365 kb
270000 0
30000
60000
90000
120000
150000
180000
210000
240000
pYEE32054P8
274.7 kb
0
30000
60000
90000
120000
150000
180000
210000
240000
pYEE32054P7
269.8 kb
0
20000
40000
60000
80000
100000
120000
140000
160000
180000
200000
pYEE32054P6
218.2 kb
pYEE32054P5
12 kb
pYEE32054P4
10.8 kb
pYEE32054P3
7.8 kb
pYEE32054P2
6.1 kb
pYEE32054P1
5.3 kb

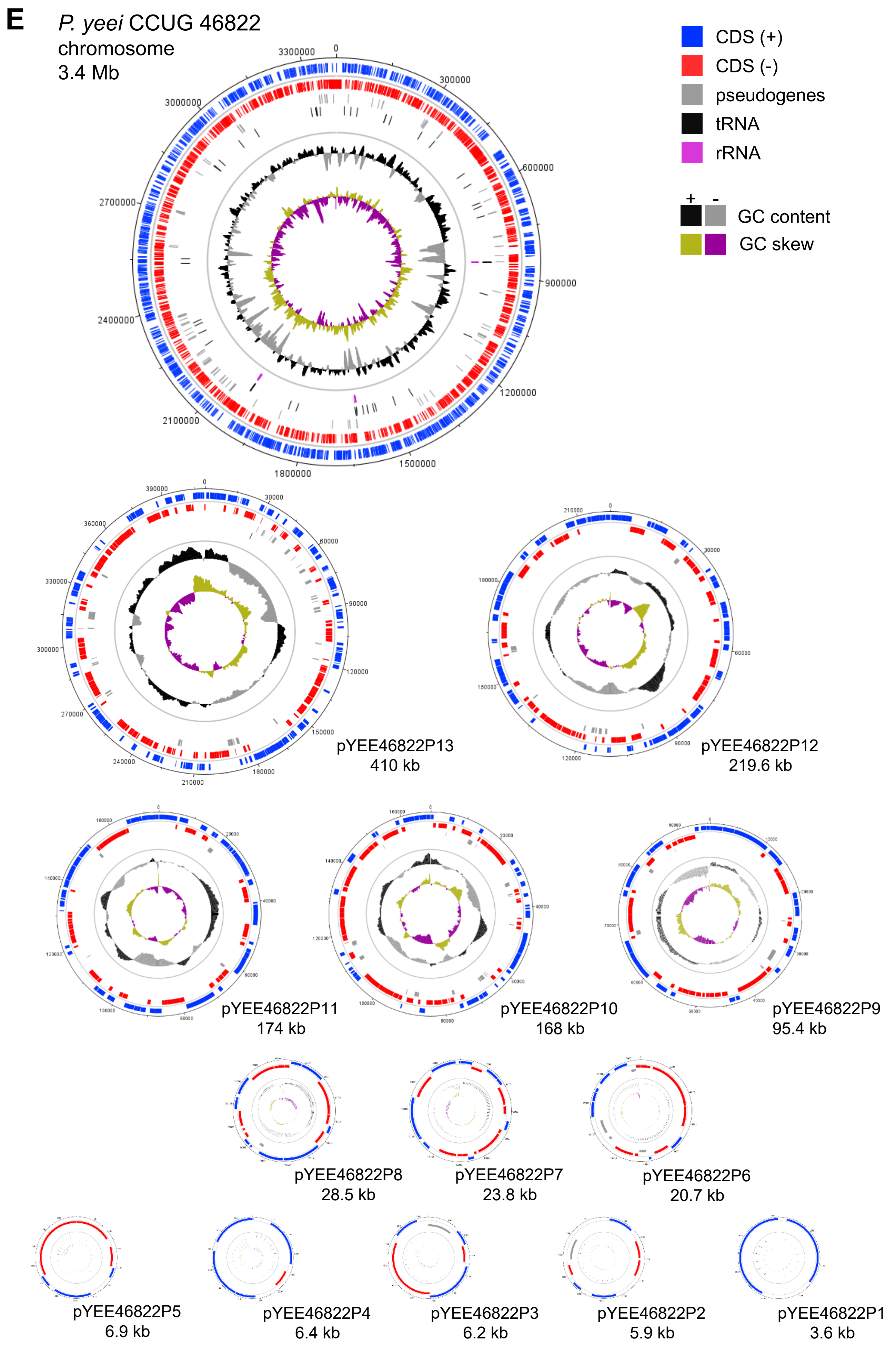
E
P. yeei CCUG 46822
chromosome
3.4 Mb
CDS (+)
CDS (-)
pseudogenes
tRNA
rRNA
+ -
GC content
GC skew
3300000
0
300000
600000
900000
1200000
1500000
1800000
2100000
2400000
2700000
3000000
pYEE46822P13
410 kb
pYEE46822P12
219.6 kb
pYEE46822P11
174 kb
pYEE46822P10
168 kb
pYEE46822P9
95.4 kb
pYEE46822P8
28.5 kb
pYEE46822P7
23.8 kb
pYEE46822P6
20.7 kb
pYEE46822P5
6.9 kb
pYEE46822P4
6.4 kb
pYEE46822P3
6.2 kb
pYEE46822P2
5.9 kb
pYEE46822P1
3.6 kb

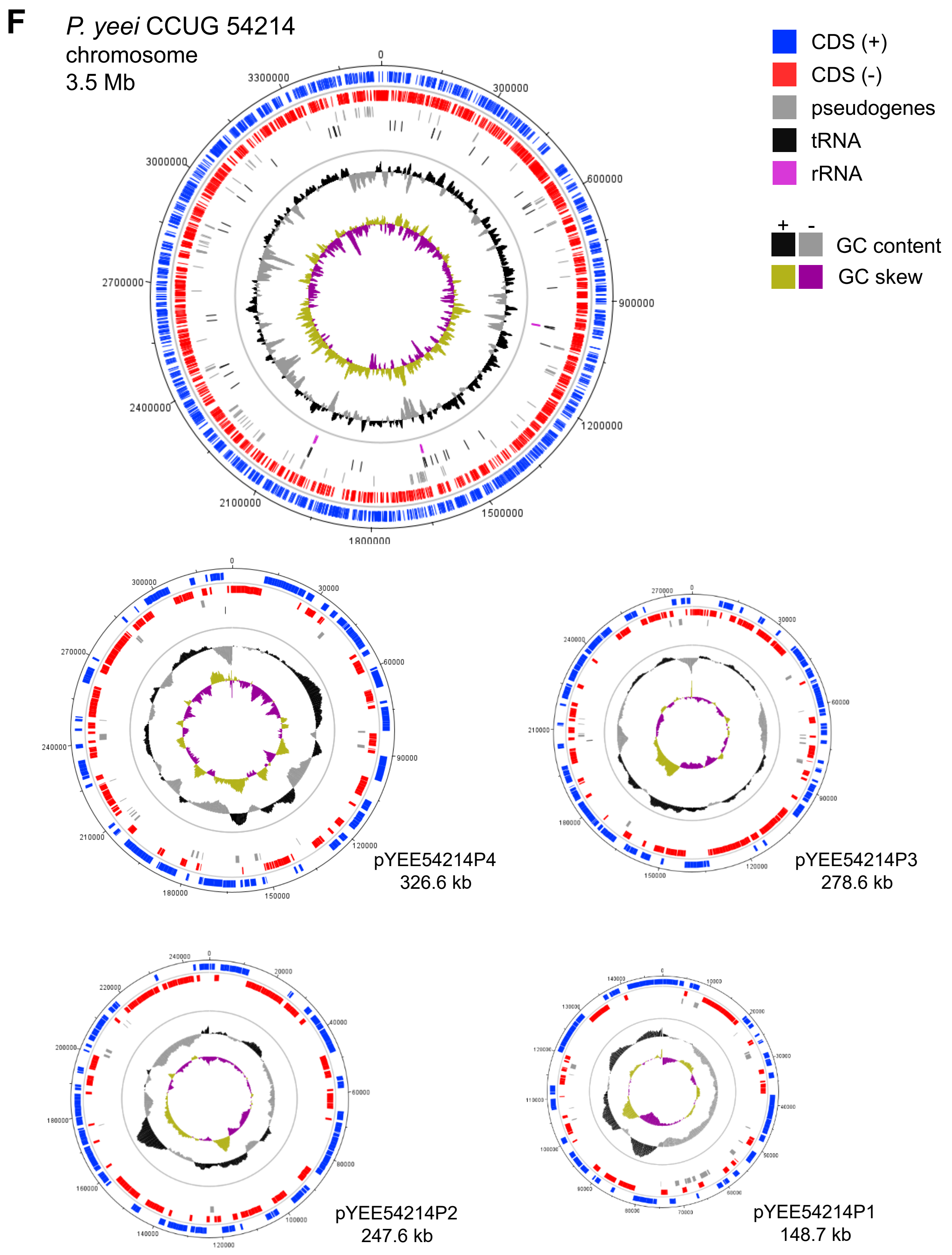
F
P. yeei CCUG 54214
chromosome
3.5 Mb
CDS (+)
CDS (-)
pseudogenes
tRNA
rRNA
+ -
GC content
GC skew
pYEE54214P4
326.6 kb
pYEE54214P3
278.6 kb
pYEE54214P2
247.6 kb
pYEE54214P1
148.7 kb

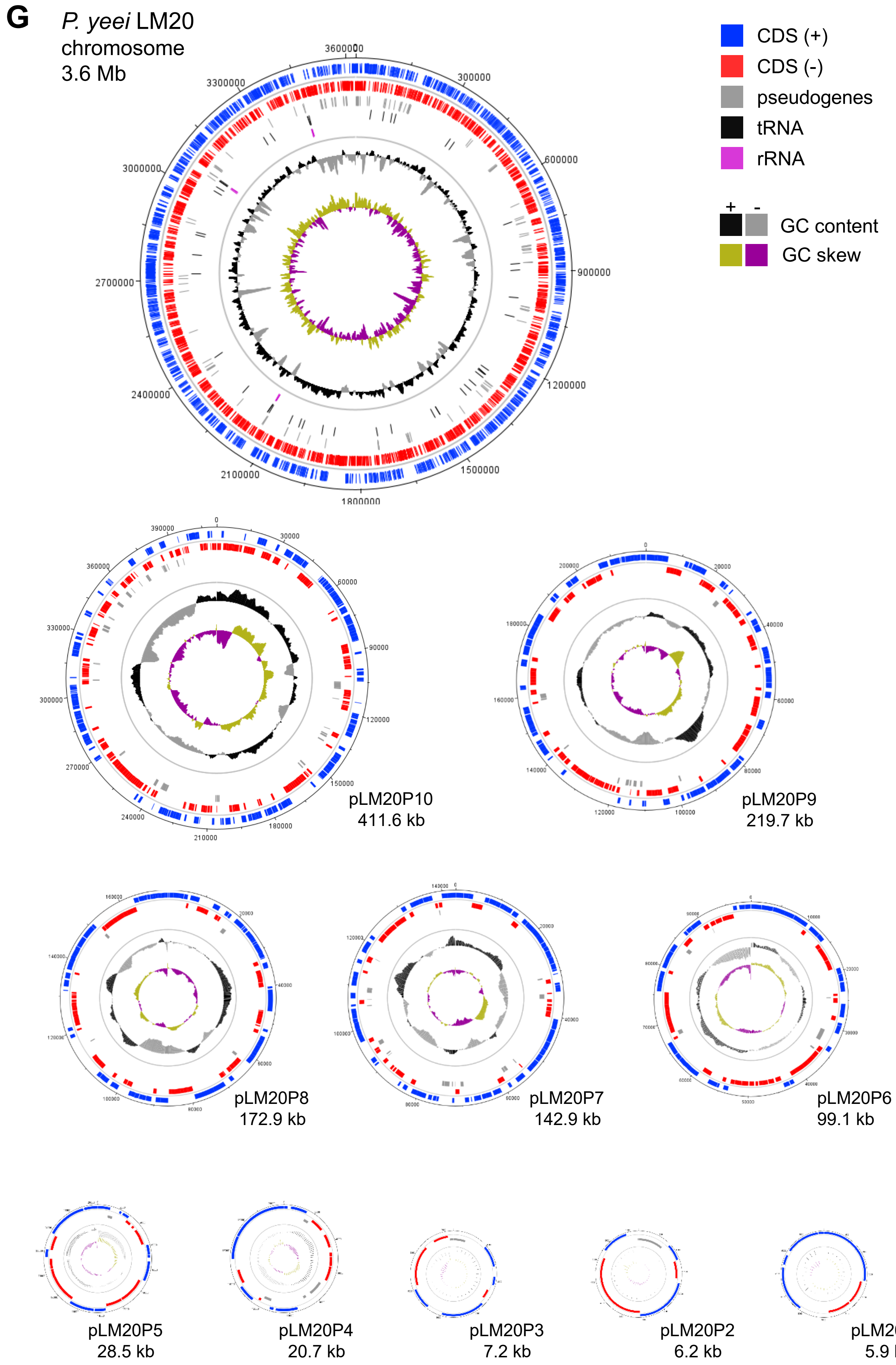
G
P. yeei LM20
chromosome
3.6 Mb
CDS (+)
CDS (-)
pseudogenes
tRNA
rRNA
+ -
GC content
GC skew
3600000
300000
600000
900000
1200000
1500000
1800000
2100000
2400000
2700000
3000000
3300000
pLM20P10
411.6 kb
pLM20P9
219.7 kb
pLM20P8
172.9 kb
pLM20P7
142.9 kb
pLM20P6
99.1 kb
pLM20P5
28.5 kb
pLM20P4
20.7 kb
pLM20P3
7.2 kb
pLM20P2
6.2 kb
pLM20P1
5.9 kb
