# Supplementary figures and images for: Genome-wide comparative analysis of clinical and environmental strains of the opportunistic pathogen Paracoccus yeei (Alphaproteobacteria)
Source: Front Microbiol. 2024 Nov 6;15:1483110. doi: 10.3389/fmicb.2024.1483110 (PMC11578231; doi:10.3389/fmicb.2024.1483110)

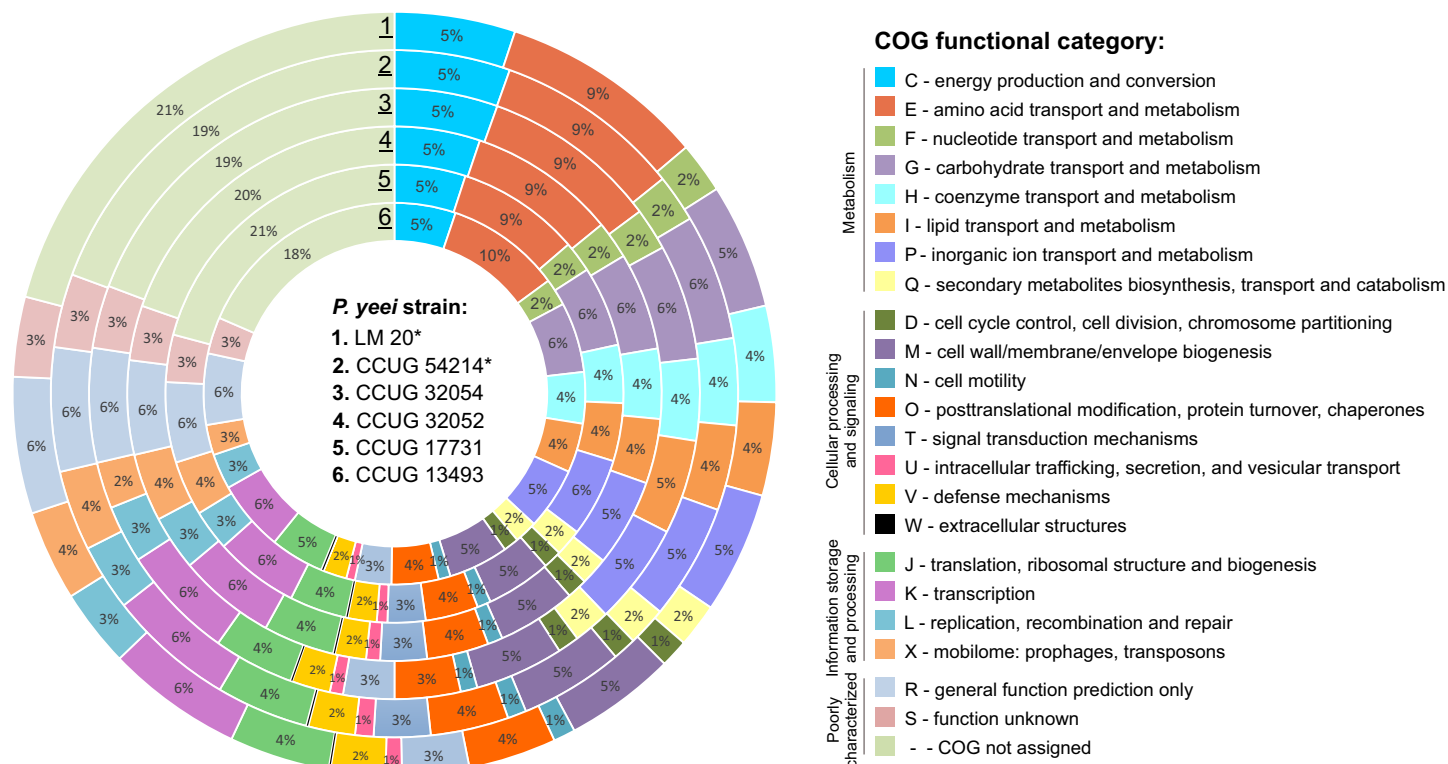

Figure S2. Distribution of COG functional categories in the analyzed *P. yeei* genomes.

Supplement: Supplementary file 2 [file Data_Sheet_2.PDF]
