# Supplementary material for: Genome-wide comparative analysis of clinical and environmental strains of the opportunistic pathogen Paracoccus yeei (Alphaproteobacteria)
Source: Front Microbiol. 2024 Nov 6;15:1483110. doi: 10.3389/fmicb.2024.1483110 (PMC11578231; doi:10.3389/fmicb.2024.1483110)
Supplement: Supplementary file 4 [file Data_Sheet_4.PDF]

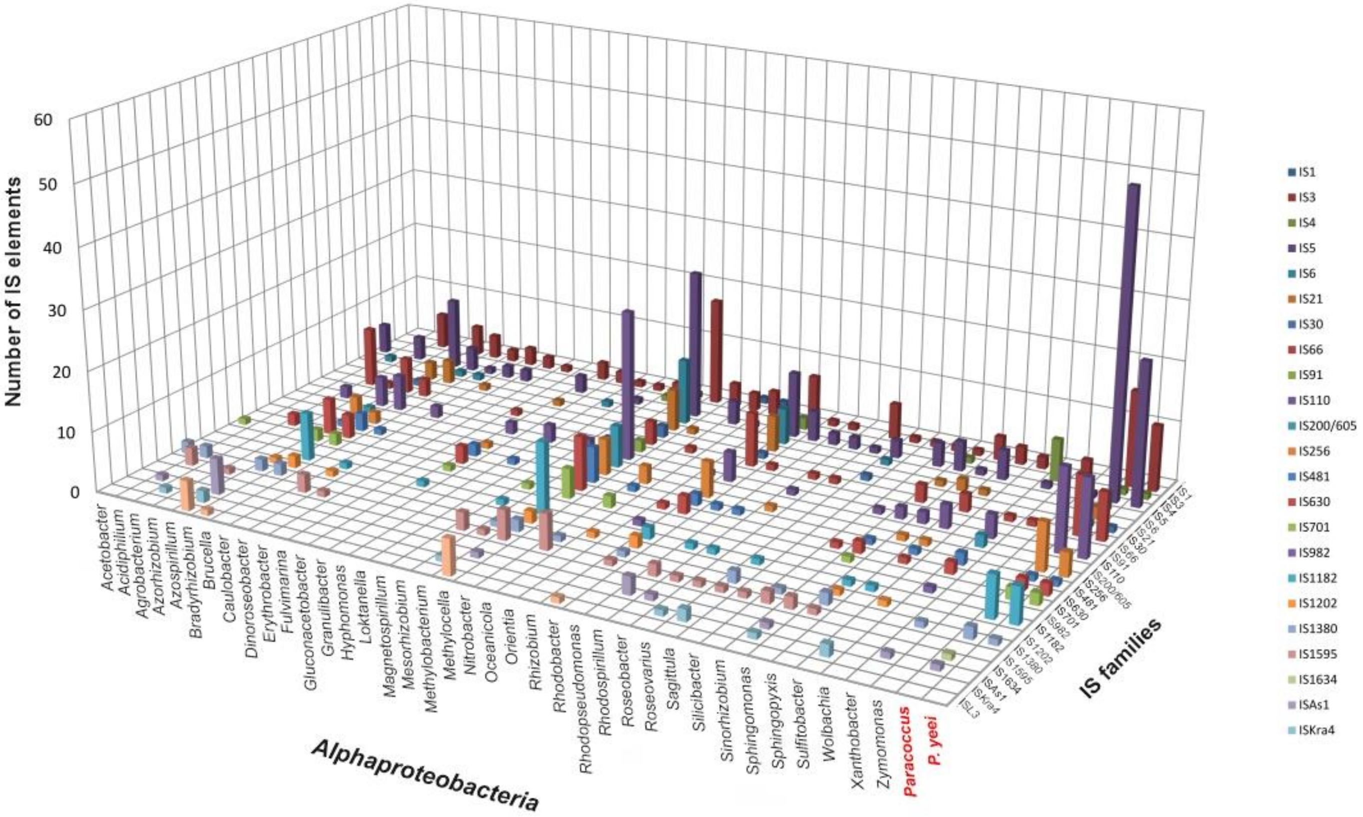

Figure S4. Distribution of IS-families identified in *Paracoccus* spp. (including *P. yeei*), among other genera of the class *Alphaproteobacteria* (ISfinder database).
