# Supplementary material for: Genome-wide comparative analysis of clinical and environmental strains of the opportunistic pathogen Paracoccus yeei (Alphaproteobacteria)
Source: Front Microbiol. 2024 Nov 6;15:1483110. doi: 10.3389/fmicb.2024.1483110 (PMC11578231; doi:10.3389/fmicb.2024.1483110)
Supplement: Supplementary file 5 [file Table_1.doc]

**TABLE S1.** Plasmids and oligonucleotide primers used in this study.

| **Plasmid or primer** | **Description or sequence (5’->3’)** | **Source or reference** |
| --- | --- | --- |
| **Plasmid** |  |  |
| pABW1 | Mobilizable cloning vector; Km^r^; *ori* pMB1; *oriT* RK2; *lacZα*; MCS | Bartosik et al., 1997 |
| pABW-m13493P1 | Km^r^; ori pMB1; ori pYEE13493P1; oriT RK2  Mobilizable E. coli-Paracoccus spp. shuttle plasmid constructed by insertion of a PCR product of fragment of pYEE13493P1 (containing the plasmid replication system; 1771 bp) into the KpnI/XbaI site of pABW1. | This study |
| pABW-m17731P1 | Km^r^; ori pMB1; ori pYEE17731P1; oriT RK2  Mobilizable E. coli-Paracoccus spp. shuttle plasmid constructed by insertion of a PCR product of fragment of pYEE17731P1 (containing the plasmid replication system; 1771 bp) into the KpnI/XbaI site of pABW1. | This study |
| pABW-m32052P2 | Km^r^; ori pMB1; ori pYEE32052P2; oriT RK2  Mobilizable E. coli-Paracoccus spp. shuttle plasmid constructed by insertion of a PCR product of fragment of pYEE32052P2 (containing the plasmid replication system; 1771 bp) into the KpnI/XbaI site of pABW1. | This study |
| pABW- m32052P3 | Km^r^; ori pMB1; ori pYEE32052P3; oriT RK2  Mobilizable E. coli-Paracoccus spp. shuttle plasmid constructed by insertion of a PCR product of fragment of pYEE32052P3 (containing the plasmid replication system; 2924 bp) into the KpnI/XbaI site of pABW1. | This study |
| pABW- m32052P6 | Km^r^; ori pMB1; ori pYEE32052P6; oriT RK2  Mobilizable E. coli-Paracoccus spp. shuttle plasmid constructed by insertion of a PCR product of fragment of pYEE32052P6 (containing the plasmid replication system; 3497 bp) into the KpnI/XbaI site of pABW1. | This study |
| pABW- m32054P1 | Km^r^; ori pMB1; ori pYEE32054P1; oriT RK2  Mobilizable E. coli-Paracoccus spp. shuttle plasmid constructed by insertion of a PCR product of fragment of pYEE32054P1 (containing the plasmid replication system; 1768 bp) into the KpnI/XbaI site of pABW1. | This study |
| pABW- m32054P2 | Km^r^; ori pMB1; ori pYEE32054P2; oriT RK2  Mobilizable E. coli-Paracoccus spp. shuttle plasmid constructed by insertion of a PCR product of fragment of pYEE32054P2 (containing the plasmid replication system; 2161 bp) into the KpnI/XbaI site of pABW1. | This study |
| pABW- m32052P4 | Km^r^; ori pMB1; ori pYEE32054P4; oriT RK2  Mobilizable E. coli-Paracoccus spp. shuttle plasmid constructed by insertion of a PCR product of fragment of pYEE32054P4 (containing the plasmid replication system; 1662 bp) into the KpnI/XbaI site of pABW1. | This study |
| pABW- mLM20P3 | Km^r^; ori pMB1; ori pLM20P3; oriT RK2  Mobilizable E. coli-Paracoccus spp. shuttle plasmid constructed by insertion of a PCR product of fragment of pLM20P3 (containing the plasmid replication system; 1524 bp) into the KpnI/XbaI site of pABW1. | This study |
| pABW- mLM20P4 | Km^r^; ori pMB1; ori pLM20P4; oriT RK2  Mobilizable E. coli-Paracoccus spp. shuttle plasmid constructed by insertion of a PCR product of fragment of pLM20P4 (containing the plasmid replication system; 1832 bp) into the KpnI/XbaI site of pABW1. | This study |
| pBBRMCS-5 | Gm^r^; *lacZα*; *ori*T RK2; | Kovach et al., 1995 |
| pBBR-*ure*α | Gm^r^; plasmid constructed by insertion of a PCR product of fragment URE type 1 (*ureαβγ*) into pBBRMCS-5, used to complement mutations *ureα*::Km^r^ in the URE module | This study |
| pBBR-*nikR* | Gm^r^; plasmid constructed by insertion of a PCR product of fragment URE type 1 (*nikR*) into pBBRMCS-5, used to complement mutations *nikR*::Km^r^ in the URE module | This study |
| pDIY-Km | Km^r^; 13,1 kpz; *ori* ColE1 | Dziewit et al., 2011 |
| pDS132 | Cm^r^; *ori* R6K; *oriT* RK2; *sacB* | Philippe et al., 2004 |
| pDS132-*ure*α | Cm^r^ Km^r^; plasmid constructed by insertion of a PCR product of fragment URE type 1 (*ureαβγ*) into the SacI/SphI site of pDS132 and the Km^r^ cassette (cut out with SmaI enzyme from pDIY-Km) inserted into the AleI site of cloned DNA fragment | This study |
| pDS132-*nikR* | Cm^r^ Km^r^; plasmid constructed by insertion of a PCR product of fragment URE type 1 (*nikR*) into the SacI/SphI site of pDS132 and the Km^r^ cassette (cut out with SmaI enzyme from pDIY-Km) inserted into the AleI site of cloned DNA fragment | This study |
| pMAT1 | Entrapment shuttle vector; Km^r^; *ori* pBBR1; *ori*T RK2; *sacB* | Szuplewska and Bartosik, 2009 |
| pMEC1 | Entrapment shuttle vector; Km^r^; *ori* ColE1; *ori* pWKS1; *oriT* RK2; *cI-tetA* | Bartosik et al., 2003 |
| pRK2013 | Km^r^; 48 kb; ori ColE1; Tra^+^ RK2 | Ditta et al., 1980 |
| **Primer** |  |  |
| Primer pairs used for the PCR amplification of *cI-tetA* specific regions of entrapment vector pMEC1 | | |
| ALIS | 5’-TTGTAATCAGCTATGCGCCG-3’ | Bartosik et al., 2003b |
| ARIS | 5’-TCTGGCTTGAGGTTGAAGGT-3’ | Bartosik et al., 2003b |
| BLIS | 5’-TGGTGCGGTCATGGAATTAC-3’ | Bartosik et al., 2003b |
| BRIS | 5’-GTATGCAGCCGTCACTTAGA-3’ | Bartosik et al., 2003b |
| CLIS | 5’-TCCCTGCCTGAACATGAGAA-3’ | Bartosik et al., 2003b |
| CRIS | 5’-ACACAAGAGCAGCTTGAGGA-3’ | Bartosik et al., 2003b |
| DLIS | 5’-TCTTGTCTGCGACAGATTCC-3’ | Bartosik et al., 2003b |
| DRIS | 5’-TTCATACACGGTGCCTGACT-3’ | Bartosik et al., 2003b |
| Primer pairs used for the PCR amplification of *sacB* specific regions of entrapment vector pMAT1 | |  |
| A489SB | 5’-CAGACCGCTAACACAGTACA-3’ | Szuplewska and Bartosik, 2009 |
| A869SB | 5’-TTAGGATCTCCGGCTAATGC-3’ | Szuplewska and Bartosik, 2009 |
| B824SB | 5’-ACTATCACGGCTACCACATC-3’ | Szuplewska and Bartosik, 2009 |
| B1253SB | 5’-TTGTCGCCTGAGCTGTAGTT-3’ | Szuplewska and Bartosik, 2009 |
| C1225SB | 5’-GATGAAGGCAACTACAGCTC-3’ | Szuplewska and Bartosik, 2009 |
| C1639SB | 5’-GACGATTGACGGCATTACGT-3’ | Szuplewska and Bartosik, 2009 |
| D1619SB | 5’-TGACGATTGACGGCATTACG-3’ | Szuplewska and Bartosik, 2009 |
| D1928SB | 5’-GACAGCATCCTTGAACAAGG-3’ | Szuplewska and Bartosik, 2009 |
| Primer pairs used PCR amplification of selected types of *P. yeei* REP modules | | |
| FmLMP4 | 5’-ATGGTACCAAGGGTGGACTTACCGACTC-3’ | This study |
| RmLMP4 | 5’-GCTCTAGAGCCAGCTGAAGGATCTCTAC-3’ | This study |
| Fm054p1 | 5’-ATCGGTACCTCCAATCCATCGGCGTCATC-3’ | This study |
| Rm054p1 | 5’-GCATCTAGAGAGGCAATCGACACCTTCAC-3’ | This study |
| Fm493p1 | 5’-ATGGTACCCTATCGGCGTCATTGTTGGG-3’ | This study |
| Rm493p1 | 5’-GCTCTAGAGAGGCAATCGACACCTTCAC-3’ | This study |
| Fm052p6 | 5’-ATGGTACCCGTCATCCCTGCCTCATTTG-3’ | This study |
| Fm052p6 | 5’-GCTCTAGAACGCCCATATGCCCGATCTC-3’ | This study |
| Fm052p2 | 5’-ATGGTACCCTATCGGCGTCATTGTTGGG-3’ | This study |
| Rm052p2 | 5’-GCTCTAGAGAGGCAATCGACACCTTCAC-3’ | This study |
| FmLMp3 | 5’-ATGGTACCACGCAGAGCAAGGACATCAG-3’ | This study |
| RmLMp3 | 5’-GCTCTAGAGGTGATAGATCGCCATCTCG-3’ | This study |
| Fm054p2 | 5’-ATCGGTACCTTAGGCCGCAAGCTGTGATG-3’ | This study |
| Rm054p2 | 5’-GCATCTAGAATTATACCGGGCCGGACTTC-3’ | This study |
| Fm052p3 | 5’-ATGGTACCTATCCGTTTCGCTGGGCTTC-3’ | This study |
| Rm052p3 | 5’-GCTCTAGAGGCGAGGGCACAATCTATTC-3’ | This study |
| Fm053p4 | 5’-GTGGTACCCGGCCTCTTCGATGATGTTC-3’ | This study |
| Rm053p4 | 5’-CGTCTAGATCCGCTATGGCGGGCATCTG-3’ | This study |
| Fm052p4 | 5’-atGAATTCGATGTGGTGCTCTGCCAGTA-3’ | This study |
| Rm052p4 | 5’-gcGGTACCCTCTCGCGCATCAGCGAGGG-3’ | This study |
| Fm052p2 | 5’-ATGGTACCCTATCGGCGTCATTGTTGGG-3’ | This study |
| Rm052p2 | 5’-GCTCTAGAGAGGCAATCGACACCTTCAC-3’ | This study |

|  |  |
| --- | --- |
