# Supplementary material for: Genome-wide comparative analysis of clinical and environmental strains of the opportunistic pathogen Paracoccus yeei (Alphaproteobacteria)
Source: Front Microbiol. 2024 Nov 6;15:1483110. doi: 10.3389/fmicb.2024.1483110 (PMC11578231; doi:10.3389/fmicb.2024.1483110)
Supplement: Supplementary file 6 [file Table_2.doc]

**Table S2. Minimal inhibitory concentrations (MICs) of heavy metals and antibiotics.**

| **Heavy metal/**  **Antibiotic** | ***P. yeei clinical isolates*** | | |  | ***environmental isolates*** | | |
| --- | --- | --- | --- | --- | --- | --- | --- |
|  | **CCUG 13493** | **CCUG 17731** | **CCUG 32052** | **CCUG 32054** | **CCUG 46822** | **CCUG 54214** | **LM20**^1^ |
|  | ***Minimum inhibitory concentration (MIC) of heavy metals [mM]:*** | | | | | | |
| As^3+^ | 4 | 5 | 0.4 | 4 | 0.2 | 6 | 6 |
| Cd^2+^ | 0.3 | 0.8 | 0.3 | 0.3 | 0.2 | 0.4 | 1 |
| Co^2+^ | 2 | 1.5 | 0.7 | 0.7 | 4 | 0.7 | 1.5 |
| Cr^6+^ | 0.2 | 0.3 | 0.2 | 0.3 | 0.2 | 0.3 | 0.1 |
| Cu^2+^ | 3 | 4 | 3 | 3 | 4 | 4 | 3 |
| Hg^2+^ | 0.02 | 0.02 | 0.02 | 0.02 | 0.02 | 0.02 | 0.08 |
| Ni^2+^ | 2 | 2 | 2 | 2 | 2 | 4 | 2 |
| V^5+^ | 3 | 150 | 150 | 150 | 15 | 150 | 5 |
| Zn^2+^ | 2 | 2 | 2 | 1.5 | 1.5 | 3 | 1 |
|  | ***Minimum inhibitory concentration (MIC) of antibiotics:*** | | | | | | |
| Ampicillin (AMP) | 0.064 | 2 | 0.19 | 6 | 0.38 | 0.047 | 0.19 |
| Ciprofloxacin (CIP) | 0.38 | 0.5 | 1 | 1 | 1 | 0.19 | 1 |
| Erythromycin (ERY) | 2 | 3 | 8 | 8 | 4 | 1 | 6 |
| Gentamicin (GEN) | 1 | 2 | 2 | 0.75 | 3 | 2 | 1.5 |
| Tetracycline (TET) | 0.064 | 0.25 | 0.75 | 1.7 | 0.38 | 0.094 | 0.5 |
| Vancomycin (VAN) | 3 | 4 | 1 | 8 | 3 | 1 | 2 |

^1^MICs of heavy metals for LM20 were published in Dziewit et al., 2015.

Isolates that demonstrated growth at the following minimum metal ion concentrations were classified as resistant using criteria described previously (Dziewit et al., 2015): (i) 20 mM V^5+^, (ii) 1 mM As^3+^, Cd^2+^, Co^2+^, Cu^2+^, Ni^2+^, Zn^2+^, or Cr^6+^, and (iv) 0.1 mM Hg^2+^.
