# Supplementary material for: Genome-wide comparative analysis of clinical and environmental strains of the opportunistic pathogen Paracoccus yeei (Alphaproteobacteria)
Source: Front Microbiol. 2024 Nov 6;15:1483110. doi: 10.3389/fmicb.2024.1483110 (PMC11578231; doi:10.3389/fmicb.2024.1483110)
Supplement: Supplementary file 3 [file Data_Sheet_3.PDF]

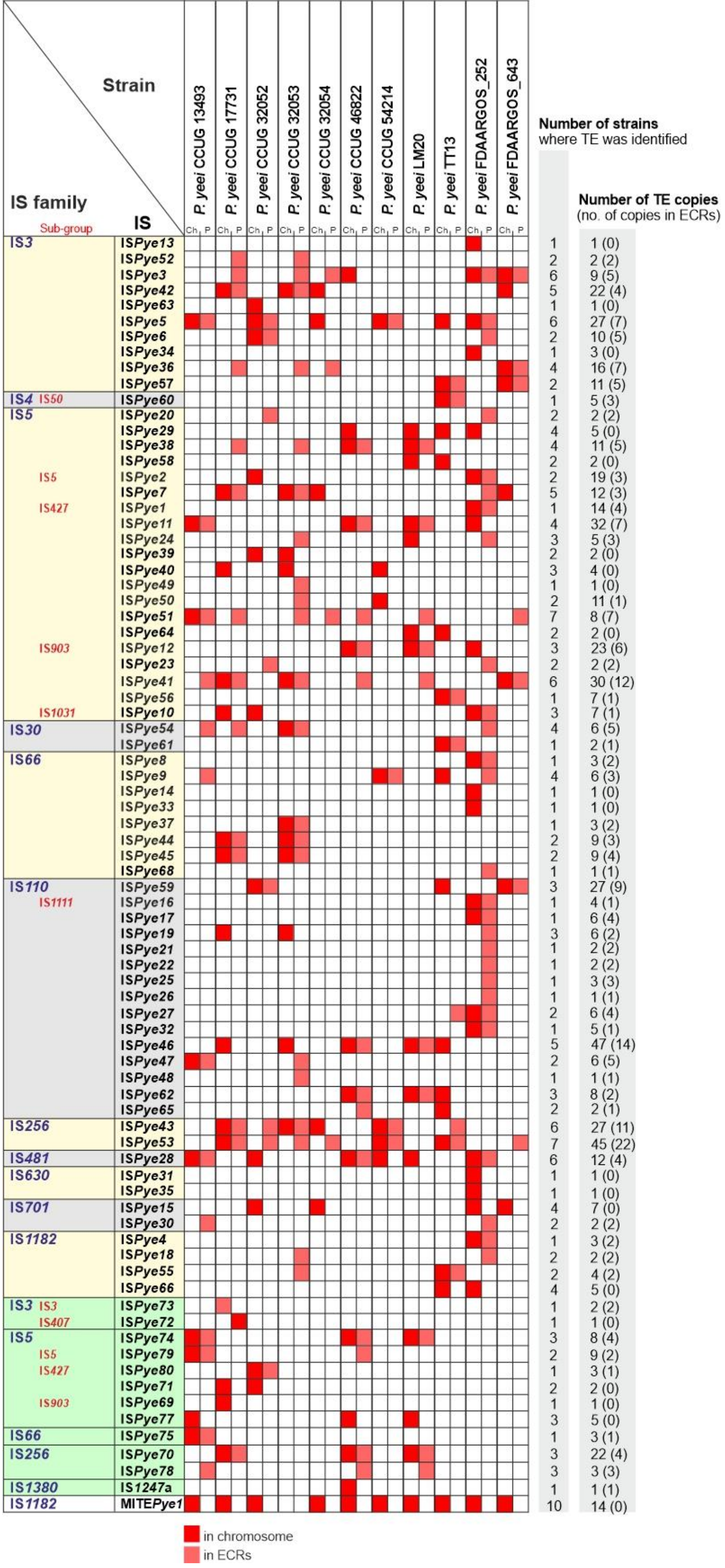

Figure S3. The distribution of all complete insertion sequeneces IS*Pye1*-IS*Pye80* of *P. yeei* (ISfinder and this study) and MITE*Pye1* element across all *P. yeei* genomes. A specific DNA probe (fragment of a TNPase gene or MITE element) was used for an *in silico* analysis. Only complete sequences or their isoforms were marked. The number of ISs present in chromosomes (Ch) and extrachromosomal replicons (P) is indicated.
